# Marine Invertebrate Extracts Induce Colon Cancer Cell Death via ROS-Mediated DNA Oxidative Damage and Mitochondrial Impairment

**DOI:** 10.3390/biom9120771

**Published:** 2019-11-23

**Authors:** Verónica Ruiz-Torres, Celia Rodríguez-Pérez, María Herranz-López, Beatriz Martín-García, Ana-María Gómez-Caravaca, David Arráez-Román, Antonio Segura-Carretero, Enrique Barrajón-Catalán, Vicente Micol

**Affiliations:** 1 Instituto de Biología Molecular y Celular (IBMC) and Instituto de Investigación, Desarrollo e Innovación en Biotecnología Sanitaria de Elche (IDiBE), Universitas Miguel Hernández (UMH), 03202 Elche, Spain; vruiz@umh.es (V.R.-T.); mherranz@umh.es (M.H.-L.); vmicol@umh.es (V.M.); 2 Department of Analytical Chemistry, Faculty of Sciences, University of Granada, 18071 Granada, Spaindarraez@ugr.es (D.A.-R.); ansegura@ugr.es (A.S.-C.); 3 Research and Development of Functional Food Centre (CIDAF), PTS Granada, Edificio BioRegion, 18016 Granada, Spain; 4 CIBER, Fisiopatología de la Obesidad y la Nutrición, CIBERobn, Instituto de Salud Carlos III., Palma de Mallorca 07122, Spain

**Keywords:** marine invertebrate, soft coral, holothurian, nudibranch, antiproliferative, colon cancer, ROS, DNA damage, cell cycle, apoptosis, necrosis, HPLC-ESI-TOF-MS, cell death, natural compounds

## Abstract

Marine compounds are a potential source of new anticancer drugs. In this study, the antiproliferative effects of 20 invertebrate marine extracts on three colon cancer cell models (HGUE-C-1, HT-29, and SW-480) were evaluated. Extracts from two nudibranchs (*Phyllidia varicosa*, NA and *Dolabella auricularia*, NB), a holothurian (*Pseudocol ochirus violaceus*, PS), and a soft coral (*Carotalcyon* sp., CR) were selected due to their potent cytotoxic capacities. The four marine extracts exhibited strong antiproliferative effects and induced cell cycle arrest at the G2/M transition, which evolved into early apoptosis in the case of the CR, NA, and NB extracts and necrotic cell death in the case of the PS extract. All the extracts induced, to some extent, intracellular ROS accumulation, mitochondrial depolarization, caspase activation, and DNA damage. The compositions of the four extracts were fully characterized via HPLC-ESI-TOF-MS analysis, which identified up to 98 compounds. We propose that, among the most abundant compounds identified in each extract, diterpenes, steroids, and sesqui- and seterterpenes (CR); cembranolides (PS); diterpenes, polyketides, and indole terpenes (NA); and porphyrin, drimenyl cyclohexanone, and polar steroids (NB) might be candidates for the observed activity. We postulate that reactive oxygen species (ROS) accumulation is responsible for the subsequent DNA damage, mitochondrial depolarization, and cell cycle arrest, ultimately inducing cell death by either apoptosis or necrosis.

## 1. Introduction

Colorectal cancer (CRC) is one of the most frequent causes of mortality worldwide, and approximately 95% of cases consist of adenocarcinoma [1]. Conventional treatment methods, such as surgery, radiation, and chemotherapy, usually fail to cure cancer in the advanced stages. For this reason, new compounds that may be suitable for the development of more active and specific treatments and are devoid of the side effects associated with conventional methods must be identified [2].

Natural products are produced by the secondary metabolism of living organisms and present great chemical diversity [3]. These products represent an almost unlimited source for the identification of novel chemical structures that might serve as a basis for the development of new anticancer drugs [4]. In the last 70 years, approximately 49% of the molecules approved to treat cancer were either originally natural products or based directly on them [5]. Most natural products are derived from plants and terrestrial microorganisms [6,7]; however, the marine environment has attracted increasing attention due to the potential druggability of marine compounds and emerging technologies in bioprospection [8].

The biodiversity of the marine ecosystem has provided a wide number of new scaffolds with putative anticancer effects [9,10]. A review of the most promising bioactive marine compounds discovered between 1985 and 2012 reported that considerable efforts have been focused on the discovery of anticancer drugs with applications in other diseases, such as cardiovascular, neuroprotective, antiviral, antifungal, and antibacterial applications [11].

At marine and coastal habitats, the environmental conditions are harder and more diverse than terrestrial due to the aquatic ecosystems along with the strong selective pressure, resulting in a much broader range of phyla and classes of organisms. For instance, coral reef ecosystems are known by their biological competition for surface and energy resources. Secondary metabolites produced in response to competitive pressure allow them to survive. Many scientific studies have shown the advantages of possessing these secondary metabolites to survive [12,13,14,15]. Of the more than 33 currently known phyla, 97% are present in and 45% are exclusive to the marine ecosystem [16]. Among marine organisms, invertebrates (animals without a backbone or spinal column) are the major source of bioactive compounds in the marine ecosystem and represent 60% of all marine animals. Marine invertebrates are members of phyla such as Porifera, Coelenterata, Mollusca, Tunicata, Annelida, and Bryozoa, and they remain the major source of anticancer compounds being tested in the preclinical phase [10,11,17]

In the present study, the potential antitumor activities of 20 extracts obtained from marine invertebrates were tested to determine their antiproliferative capacities in human colon cancer cell lines. The most promising extracts were identified from four organisms—two nudibranchs (*Phyllidia varicosa*, NA and *Dolabella auricularia*, NB), a holothurian (*Pseudocolochirus violaceus*, PS), and a soft coral (*Carotalcyon* sp., CR), and the compositions of these extracts were characterized in depth using high-performance liquid chromatography coupled to electrospray time-of-flight mass spectrometry (HPLC-ESI-TOF-MS) analysis. The reported anticancer activities of the most abundant identified compounds were reviewed to determine which compounds contributed most to the activity of the extracts. The putative molecular mechanisms of these extracts were further dissected and discussed by studying cell cycle progression, reactive oxygen species (ROS) generation, DNA damage, apoptosis, necrosis, and mitochondrial function. The results support an antiproliferative mechanism that depends on the generation of free radical species at the intracellular level.

## 2. Results

### 2.1. Marine Extracts Derived from Selected Invertebrates Inhibit the Proliferation of Colon Cancer Cells

First, 20 invertebrate marine species (Table 1) were selected as described in the methods section. Then, the cytotoxic activity of their extracts toward a panel of three human colon cancer cell lines was screened using the colorimetric cell viability assay based on the enzymatic reduction of 3-(4,5-dimethylthiazol-2-yl)-2,5-diphenyltetrazolium bromide (MTT) to MTT-formazan catalyzed by mitochondrial succinate dehydrogenase or MTT assay. Solutions of each extract were prepared at eight concentrations (0–100 µg/mL) and were used to treat HGUE-C-1, HT-29, and SW-480 cells for 24, 48, or 72 h. Survival curves were extrapolated to calculate the concentration that inhibited the growth of 50% of cells (IC_50_). These values are shown in Supplementary Table S2, and the cytotoxic curves are presented in Supplementary Figure S1. The most active extracts were defined as those with IC_50_ values less than 30 μg/mL at 48 h in at least two of the cell lines used or ≤ 15 μg/mL in at least one of the cell lines used. According to these criteria, the four extracts that presented the lowest IC_50_ values (CR from red coral, PS from a holothurian, and NA and NB from nudibranch marine organisms) were selected for further characterization. The most interesting result was obtained with NB extract, which exhibited 48-h IC_50_ values of 0.3 μg/mL (HGUE-C-1 cells), 0.1 μg/mL (HT-29 cells), and 0.6 μg/mL (SW-480 cells). Furthermore, the PS extract also showed high cytotoxicity, with IC_50_ values of 37.4 μg/mL (HGUE-C-1 cells), 0.7 μg/mL (HT-29 cells), and 18.6 μg/mL (SW-480 cells). The NA extract exhibited significant cytotoxic activity, with IC_50_ values of 137.3 μg/mL (HGUE-C-1 cells), 10.0 μg/mL (HT-29 cells), and 13.6 μg/mL (SW-480 cells), and the CR extract exhibited IC_50_ values of 82.0 μg/mL (HGUE-C-1 cells), 9.4 μg/mL (HT-29 cells), and 27.6 μg/mL (SW-480 cells) (Table 2).

**Figure 1:**
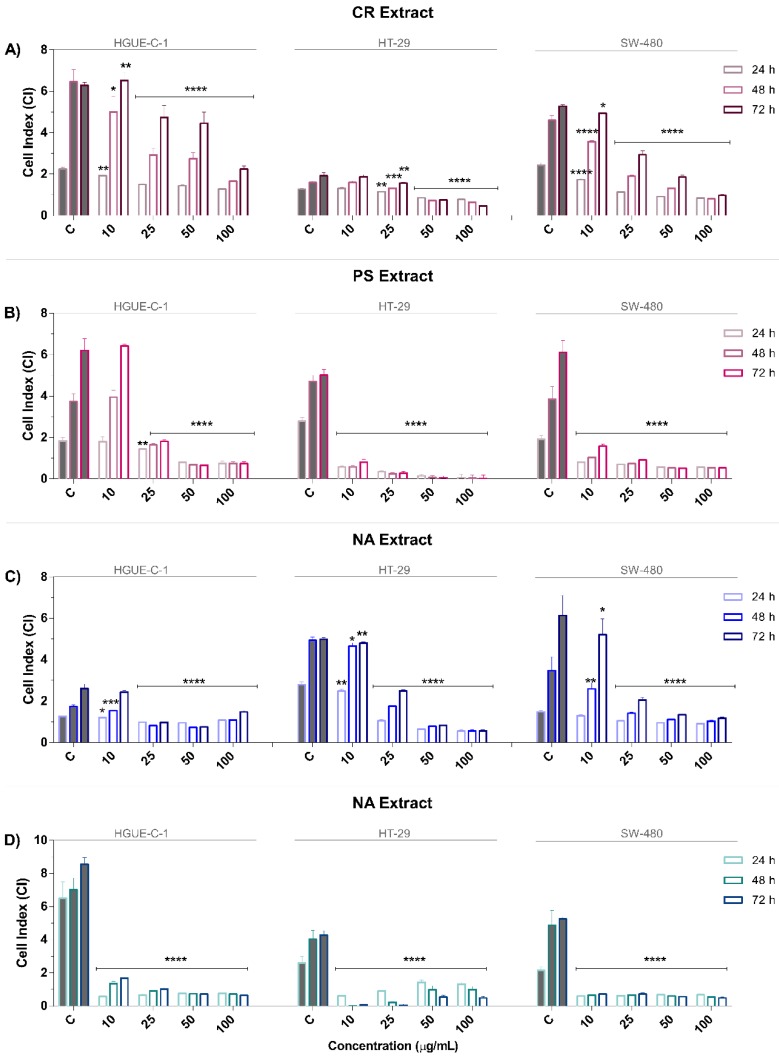
Assessment of the antiproliferative effect of invertebrate marine extracts on human colon carcinoma cell models using a real-time cell analysis (RTCA) system as described in the Materials and Methods section. HGUE-C-1, HT-29 and SW-480 cells were treated with 10, 25, 50, or 100 µg/mL *Carotalcyon* sp. (CR) (**A**), *Pseudocolochirus violaceus* (PS) (**B**), *Phyllidia varicosa* (NA) (**C**), and *Dolabella auricularia* (NB) (**D**). The CI at 24, 48, or 72 h is represented as the means ± SD of three independent experiments. *p*-values were calculated and compared to those of the respective untreated cell lines (control cells administered less than 0.2% dimethyl sulfoxide, C) using ANOVA. * *p*-value < 0.05, ** *p*-value < 0.01, *** *p*-value < 0.001, and **** *p*-value < 0.0001.

To confirm the results from the MTT assays and to explore the antiproliferative and cytostatic effects of extracts, real-time cell analysis (RTCA) was conducted for 75 h. In contrast to labor-intensive label-based end-point assays, RTCA facilitates the monitoring of the kinetics of cellular processes, such as changes in proliferation, migration and invasion [18]. This technique provides time-dependent cellular response profiles (TCRPs) that are presented as cell indexes (CIs), which are measurements of changes in the growth rate, morphology, and adhesive characteristics of the cells in culture [19]. The proliferation profiles of HGUE-C-1, HT-29, and SW-480 cells after treatment with marine extracts were analyzed (Supplementary Figures S2 (CR), S3 (PS), S4 (NA), and S5 (NB)). The CIs were calculated from the kinetic profiles at 24, 48, and 72 h and plotted in Figure 1A–D. A significant effect on the inhibition of proliferation was observed in cells treated with marine extracts compared to those treated with control. Overall, marine extracts noticeably decreased the CIs in a dose-dependent manner in all cell models used; their strong activities at lower doses is worth noting, particularly for the PS and NB extracts, which showed a dramatic decrease in the CIs directly after treatment of all cell models with the lowest concentration applied (10 μg/mL). NB substantially decreased the CI by 80.8% in HGUE-C-1 cells, 99.8% in HT-29 cells, and 86.5% in SW-480 cells at 10 μg/mL (48 h) and further enhanced that effect by 89.7% in HGUE-C-1 cells, 75.6% in HT-29 cells, and 88.9% in SW-480 cells at 100 μg/mL after 48 h of treatment (Figure 1D). PS diminished the CI by 56.8% in HGUE-C-1 cells treated with 25 μg/mL of the extract and by 81.6% at 100 μg/mL, 87.7% at 10 μg/mL, and 99.4% at 100 μg/mL in HT-29 cells after 48 h of treatment. Moreover, PS decreased the CI by 84.1% at 10 μg/mL and by 99.22% at 100 μg/mL in SW-480 cells after 48 h of treatment (Figure 1B). The CR and NA extracts decreased the proliferation and growth of all cell models examined in a dose-dependent manner, but their effects were less noticeable than those of NB and PS. After treatment with 25 or 100 μg/mL NA for 48 h, the HGUE-C-1 CI decreased 11.5% and 37.9%, the HT-29 CI decreased 5.5% and 88.7%, and the SW-480 CI decreased 25.4% and 70.0%, respectively. A remarkable effect of NA on all the cell lines tested was only observed at concentrations greater than 25 μg/mL (Figure 1C). CR was the least potent of the four extracts in reducing proliferation. Treatments with 10 μg/mL and 100 μg/mL CR reduced the CI of HGUE-C-1 cells by 7.1% and 74.4%, respectively; a 16.9% decrease in the CI at 10 μg/mL and 59.7% decrease at 100 μg/mL were observed in HT-29 cells. Finally, decreases in the CI of 22.6% and 82.6% were observed in SW-480 cells at 10 μg/mL and 100 μg/mL, respectively (Figure 1A). The RTCA results from cells treated with the marine extracts were consistent with the findings from the MTT assay and allowed us to classify the extracts from the most to the least active as follows: NB, PS, NA, and CR.

**Figure 2:**
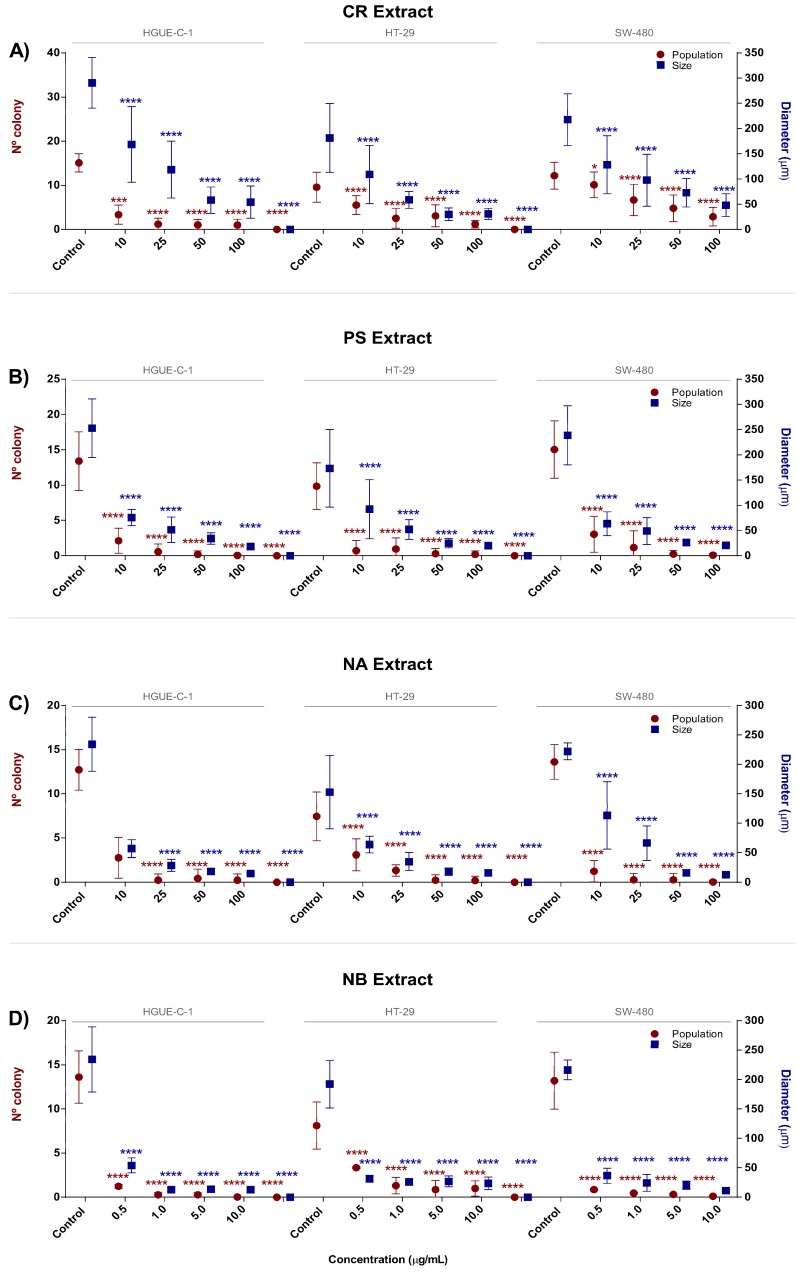
Antiproliferative effect of invertebrate marine extracts on human colon carcinoma cell colony formation. HGUE-C-1, HT-29, and SW-480 cells were treated with 0.5, 1.0, 5.0, or 10.0 µg/mL of CR (**A**), PS (**B**), NA (**C**), and NB (**D**). The population and size data from the colony formation assays (determined using Hoechst 33342) are presented as the means ± SD of three independent experiments. *p*-values were calculated and compared to those of the respective untreated cell lines (control cells administered less than 0.2% dimethyl sulfoxide, C) using ANOVAs. * *p*-value < 0.05, ** *p*-value < 0.01, *** *p*-value < 0.001, and **** *p*-value < 0.0001.

The antiproliferative activities of the selected extracts was further characterized using the colony formation assay, a technique used to measure the ability of cancer cells to proliferate after a treatment and form a large colony or a clone [20]. Marine extracts markedly reduced the size and number of colonies formed by HGUE-C-1, HT-29 and SW-480 cells, as shown in Figure 2A–D and Supplementary Figure S6. Treatment with the marine extracts decreased the average size of the colonies and the number of cells present in each colony. CR gradually reduced the average colony size in a dose-dependent manner (Figure 2A). Treatment with 100 μg/mL CR reduced the size of HGUE-C-1 cell colonies from 260 μm (14 cells per colony) to 36 μm, i.e., a 93% reduction; from 160 μm (nine cells per colony) to 24 μm (one cell per colony) in HT-29 cells, i.e., an 87% reduction; and from 226 μm (14 cells per colony) to 38 μm (two cells per colony) in SW-480 cells, i.e., a 76% decrease. Nevertheless, PS and NA were more active than CR and produced a more drastic decrease in the colony size at the lowest concentrations tested in some of the cell lines, reaching an approximately 99% reduction in the colony size of all cell lines at 100 μg/mL (Figure 2B,C). By contrast, NB produced the strongest decreases in both colony size and cell population at the lowest concentration in all cell lines tested (Figure 2D). A general reduction in the average colony size of each colon cancer cell model was induced by all the marine extracts. Although the CR extract reduced size and number in a progressive manner, the NA, PS, and NB extracts produced drastic changes in these parameters at the lowest concentrations tested.

### 2.2. Invertebrate Marine Extracts Induce Cell Cycle Arrest and Apoptosis

**Figure 3:**
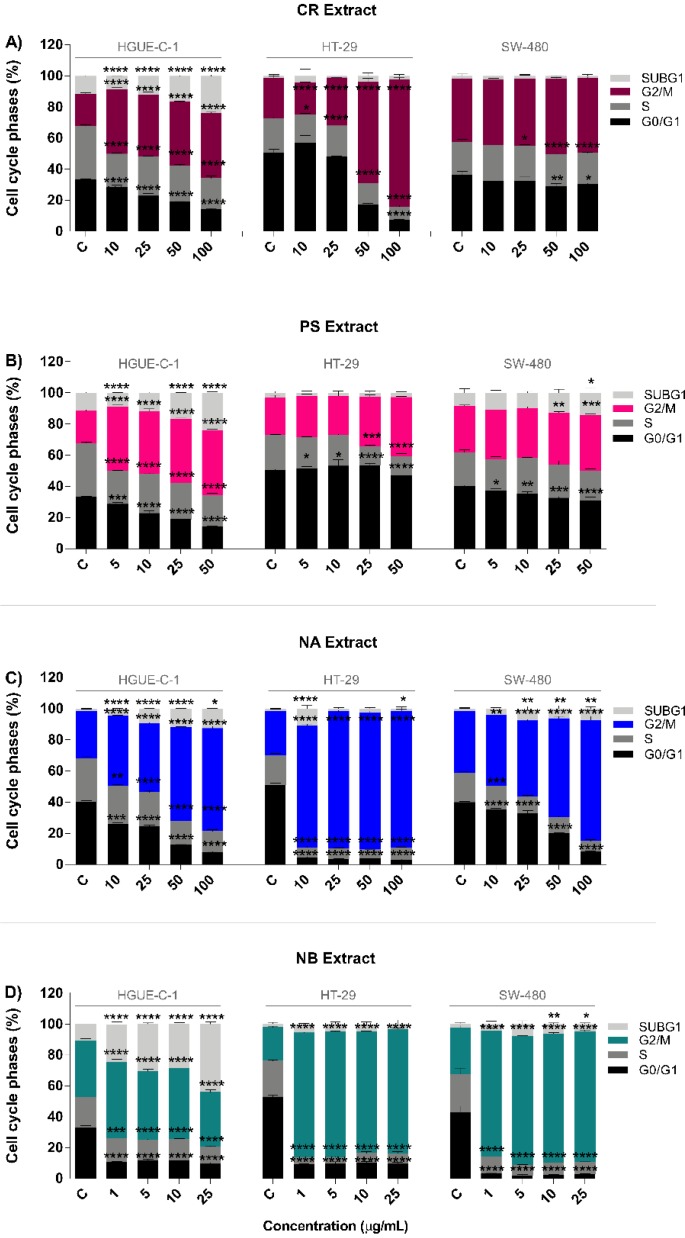
Effects of the CR (**A**), PS (**B**), NA (**C**), and NB (**D**) marine extracts on the cell cycle distribution of human colon carcinoma cell lines as assessed with a Muse^®^ Cell Analyzer. HGUE-C-1, HT-29, and SW-480 cells were plated at a density of 1 × 10^5^ cells/well, treated with different concentrations of marine extracts for 24 h, and harvested for each protocol, as described in the Materials section. The proportions of cells in the SubG1, G2/M, S, and G0/G1 phases are presented as the means ± SD of three independent experiments. *p*-values were calculated and compared to the corresponding untreated cell line (control cells administered less than 0.2% dimethyl sulfoxide, C) using ANOVA. * *p*-value < 0.05, ** *p*-value < 0.01, *** *p*-value < 0.001, and **** *p*-value < 0.0001.

After confirming the antiproliferative activity of the four selected marine extracts, the next step was to study whether these changes were mediated by a cytotoxic or cytostatic effect. Therefore, the cell cycle progression was examined using flow cytometry. After the treatment of colon cancer cells (HGUE-C-1, HT-29, and SW-480) with different concentrations of marine extracts for 24 h, alterations in the distribution of cells throughout the cell cycle were found for all combinations of extracts and cell lines (Figure 3A–D). Some differences in the pattern of phases of the cell cycle were observed depending on the extract and cell line; however, the most consistent effect was the considerable increase in the number of cells arrested at G2/M phase in all the cell lines and, in some cases, a concomitant increase in the number of cells in SubG1 phase.

At the minimum doses tested, NA and NB drastically increased the proportion of HT-29 and SW-480 cells in G2/M phase at the expense of the numbers of cells in S phase and G0/G1 phase (Figure 3C,D). In particular, the NB extract increased the proportion of HT-29 cells in G2/M phase from 21.7 ± 0.7% to 80.4 ± 0.8% at 25 μg/mL and from 30.2 ± 1.3% to 84.6 ± 0.8% in SW-480 cells. In HGUE-C-1 cells, NB increased the proportion of cells in G2/M phase in a dose-dependent manner, with a maximum percentage of 45.9 ± 0.8 observed in cells treated with 10 μg/mL, but this treatment also increased the proportion of cells in SubG1 phase (related to apoptotic cells) in a dose-dependent manner (maximum at 25 μg/mL, reaching 43.9 ± 1.5%). This increase in the proportion of cells in SubG1 phase was also observed, to a lesser extent, in HT-29 and SW-480 cells (Figure 3D).

The CR extract produced a gradual, dose-dependent change in the distribution of cells in the cell cycle for all cell lines tested. An increase in the proportion of cells in G2/M phase was also observed, particularly in HT-29 and HGUE-C-1 cells, which occurred at the expense of the proportions of cells in G0/G1 phase and S phase (Figure 3A). After treatment with 100 μg/mL CR, the proportion of cells in G2/M phase increased from 20.8 ± 0.6% to 41.7 ± 0.6% in HGUE-C-1 cells, from 25.9 ± 1.3% to 81.7 ± 1.7 in HT-29 cells, and from 40.5 ± 0.4% to 48.2 ± 0.6% in SW-480 cells. Interestingly, a significant, approximately two-fold increase in the proportion of HGUE-C-1 cells in SubG1 phase (cells in apoptosis) was observed following treatment with the CR extract, similar to that observed after treatment with the NA and NB extracts. This increase was also observed, but to a lesser extent, in HT-29 cells.

The PS extract also induced G2/M arrest in all cell lines tested; however, the change was not as prominent as that observed for the NB, NA, and CR extracts. The percentage of HGUE-C-1 cells and, to a lesser extent, SW-480 cells treated with PS extract in SubG1 phase also increased (Figure 3B).

To further study the effect of marine extracts on the induction of apoptosis in colon cancer cell models (HGUE-C-1, HT-29, and SW-480), the Annexin V detection method was used, which allowed us to discriminate early apoptosis (cells expressing phosphatidylserine on the outer leaflet of the plasma membrane) from late apoptosis and necrosis (cells that have lost cell membrane integrity and are permeable to vital dyes). Nevertheless, this assay is not able to distinguish cells in the late apoptotic state and necrotic cells because they will be positive for both Annexin V and the vital dye. To distinguish necrotic cells and late apoptotic cells, a time course experiment monitoring the shift of cells from early apoptosis to late apoptosis would be required [21,22].

**Figure 4:**
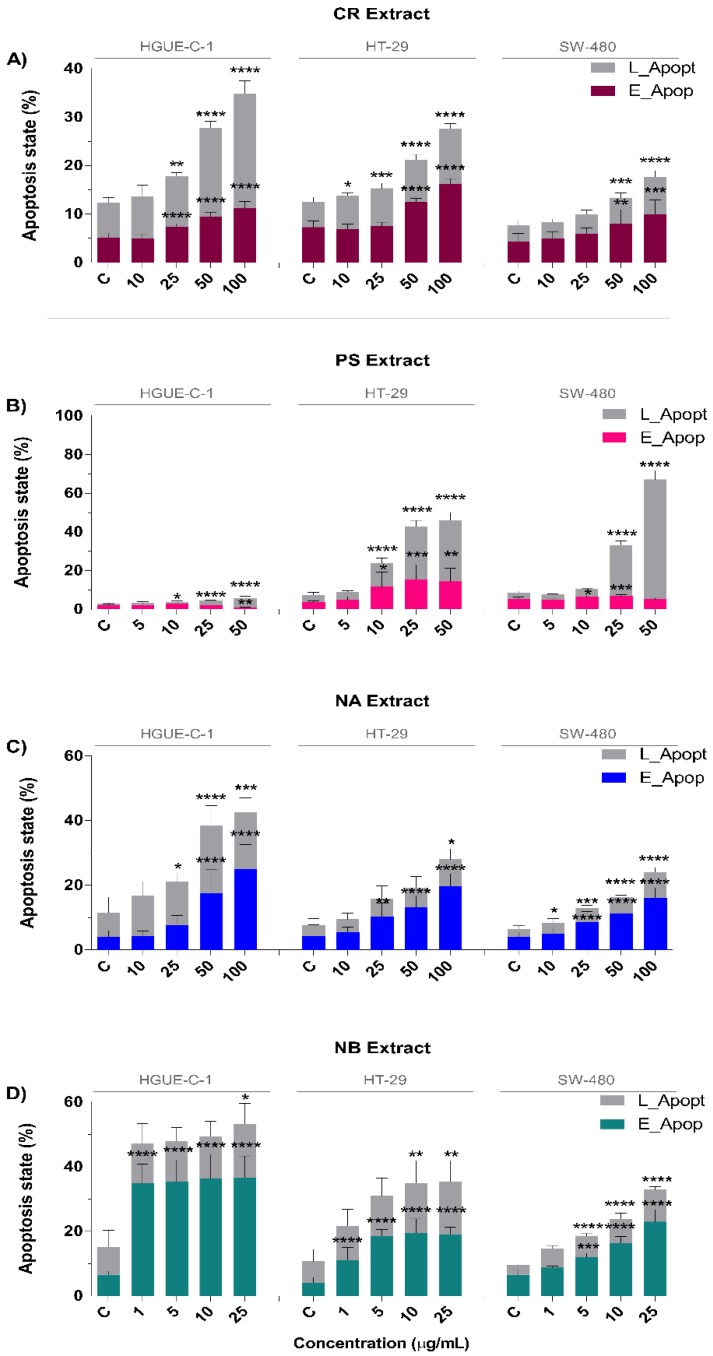
Effects of the CR (**A**), PS (**B**), NA (**C**), and NB (**D**) marine extracts on apoptosis of human colon carcinoma cell lines as measured with a Muse^®^ Cell Analyzer. HGUE-C-1, HT-29, and SW-480 cells were plated at a density of 1 × 10^5^ cells/well and treated with different concentrations of marine extracts for 24 h as described in the Materials section. The proportions of early apoptotic (E_Apop) and late apoptotic (L_Apop) cells are presented as the means ± SD of three independent experiments. *p*-values were calculated and compared to those of corresponding untreated cells (control cells administered less than 0.2% dimethyl sulfoxide, C) using ANOVAs. * *p*-value < 0.05, ** *p*-value < 0.01, *** *p*-value < 0.001, and **** *p*-value < 0.0001.

Figure 4A–D shows the effects of marine extracts on apoptosis as assessed by Annexin V treatment; the extracts were classified into two groups. The CR, NA, and NB extracts exerted a similar effect, i.e., an increase in the total number of apoptotic cells, along with a higher proportion of early apoptotic cells than late apoptotic cells. This effect was dose-dependent, indicating that the apoptotic pathway was induced in these cells. By contrast, PS increased the percentage of late apoptotic cells compared with early apoptotic cells, suggesting a potential necrotic mechanism. In more detail, treatment with 100 μg/mL CR increased the proportion of early apoptotic cells two-fold compared to that of untreated HGUE-C-1, HT-29, and SW-480 cells. The CR extract also showed a two-fold increase in the proportion of late apoptotic HT-29 and SW-480 cells and a three-fold increase in the proportion of late apoptotic HGUE-C-1 cells. Only in the case of HGUE-C-1 cells was the proportion of late apoptotic cells higher than that of early apoptotic cells (Figure 4A). Treatment with 100 μg/mL NA increased the proportions of early apoptotic HGUE-C-1, HT-29, and SW-480 cells by 6-fold, 4.5-fold and 4-fold, respectively. The proportions of late apoptotic HGUE-C-1, HT-29, and SW-480 cells were also increased 2.3-fold, 2.6-fold, and 3.3-fold, respectively (Figure 4C). Compared to no treatment, NB (100 μg/mL) increased the proportions of early apoptotic HGUE-C-1, HT-29, and SW-480 cells by 5.5-fold, 4.6-fold, and 3.5-fold, respectively, and of late apoptotic HGUE-C-1, HT-29, and SW-480 cells by 2.0-fold, 2.5-fold, and 3.4-fold, respectively (Figure 4D). Finally, compared to no treatment, PS reduced the proportions of early apoptotic HGUE-C-1 and SW-480 cells and increased the proportion of early apoptotic HT-29 cells 3-fold. By contrast, the PS extract noticeably increased the proportions of late apoptotic HGUE-C-1, HT-29, and SW-480 cells by 5.2-fold, 8.6-fold, and 18.8-fold, respectively (Figure 4B).

### 2.3. Effect of Marine Extracts on the Nonapoptotic Cell Death of Colon Cancer Cells

**Figure 5:**
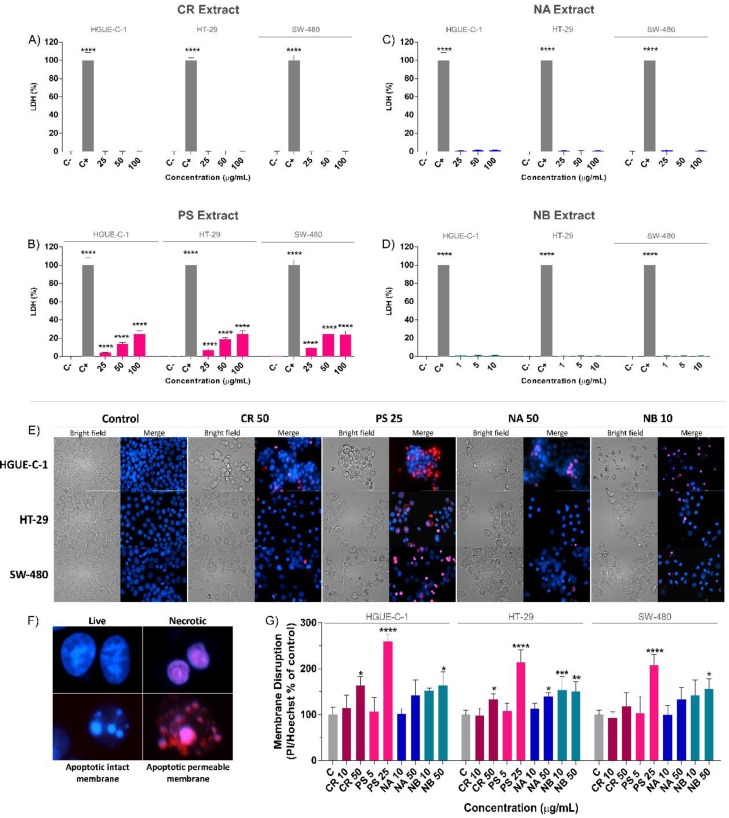
Effects of the marine extracts on lactate dehydrogenase (LDH) release (**A**–**D**) and the formation of apoptotic bodies in colon cancer cells (**E**–**G**). The effects of the invertebrate marine extracts CR (**A**), PS (**B**), NA (**C**), and NB (**D**) on LDH release from colon cancer cells after 24 h of treatment were determined as described in the Materials and Methods section and compared to the LDH release from untreated cells (control cells administered less than 0.2% dimethyl sulfoxide, C). The results are presented as the percentages (means ± SD) from three independent experiments. *p*-values were calculated and compared to the same untreated cell line using ANOVAs. * *p*-value < 0.05, ** *p*-value < 0.01, *** *p*-value < 0.001, and **** *p*-value < 0.0001. The morphology of colon cancer cells treated with marine extracts for 24 h was examined under a fluorescence microscope after staining with Hoechst 33342 and propidium iodide (PI). Representative fluorescence microscopy images of HGUE-C-1, HT-29, and SW-480 cells treated with the different marine extracts shown in bright field, DAPI (Hoechst) and Texas red (PI) channels at 20X (**E**). Morphological classification of the nuclei in live cells, necrotic cells, and apoptotic cells with intact membranes and permeable membranes (**F**). Quantification of membrane disruption (PI/Hoechst ratio) as an indicator of necrosis of colon cancer cells treated with various concentrations of the different marine extracts (**G**). The results are presented as the percentages (means ± SD) from three independent experiments. *p*-values were calculated and compared to the same untreated cell line using ANOVAs. * *p*-value < 0.05, ** *p*-value < 0.01, *** *p*-value < 0.001, and **** *p*-value < 0.0001.

Lactate dehydrogenase (LDH) is one of the most abundant cytoplasmic enzymes that is released into the extracellular space when the plasma membrane is disrupted during necrotic cell death. LDH release was measured in the three colon cancer cell lines (HGUE-C-1, HT-29, and SW-480) after treatment with different concentrations of the marine extracts (CR, PS, NA and NB) for 24 h and was compared to that of cells treated with the positive control (C+, 100% LDH activity) and negative control (C-, 0% LDH activity) (Figure 5A–D). Among the four marine extracts, the PS extract was the only extract that significantly increased LDH release compared with the negative control. Compared to the negative control, PS increased LDH release in cells from all tested cell lines by > 24%. The percentage of LDH release induced by 100 µg/mL PS after 24 h of treatment was 24.71 ± 3.16% in HGUE-C-1 cells, 24.80 ± 3.16% in HT-29 cells, and 24.14 ± 3.54% in SW-480 cells (Figure 5B). Based on these results, PS seemed to induce cell death through a necrosis-related mechanism, as suggested by the late apoptosis results described above. By contrast, the cell death induced by the other extracts (CR, NB, and NA) appeared to be mediated by the apoptotic pathway.

To further characterize the cell death process of colon cancer cells exposed to marine extracts, Hoechst/propidium iodide nuclear staining and fluorescence microscopy were performed. The cells were co-stained with the exclusion dye propidium iodide, which does not cross the intact plasma membrane, and the nuclear dye Hoechst 33342. Morphological changes were monitored using a high-content imaging fluorescent cell analyzer. The cells were classified according to the shape of the nuclei and the ratio of propidium iodide (PI) (red) and Hoechst (blue) staining (Figure 5F). The cells were considered viable if they presented a round blue nucleus, necrotic cells exhibited round purple nuclei, and apoptotic cells showed characteristic apoptotic bodies in pink when the membrane was permeabilized and in blue when the membrane remained intact (Figure 5F). Representative microscope images were taken after cells were treated with marine extracts (Figure 5E). After treatment with NB, NA or CR for 24 h, the results showed significant and common phenomena related to apoptosis, such as membrane blebbing, pyknosis and apoptotic bodies. NB and NA induced the formation of well-characterized and evident apoptopodia (apoptotic membrane protrusions) in SW-480 cells, and apoptotic bodies with intact and permeable membranes were detected in all the cell lines examined (Figure 5E). All these features were commonly observed during apoptosis. By contrast, PS induced the formation of round pink nuclei in all cells from each of the cell lines tested, indicating rapid permeabilization and disruption of the cell membrane, which is characteristic of the necrosis process.

The fluorescence intensities of PI and Hoechst 33342 were measured and normalized to those of untreated cells. A higher PI/Hoechst ratio indicates a greater degree of disruption of the plasma membrane and a higher probability of the occurrence of necrosis (Figure 5G). The PS extract significantly increased the PI/Hoechst ratio by 2.6-fold in HGUE-C-1 cells, 2.1-fold in HT-29 cells, and 2.1-fold in SW-480 cells. The CR and NB extracts increased the intensity of PI at higher concentrations; however, the significance of the difference was less than that elicited by the PS extract. These results corroborate the observations from the LDH release assay. While NB, NA, and CR appeared to induce cell death through an apoptotic mechanism, PS appeared to induce cell death via necrosis.

### 2.4. Marine Extracts Promote Intracellular ROS Generation and Mitochondrial Membrane Depolarization

**Figure 6:**
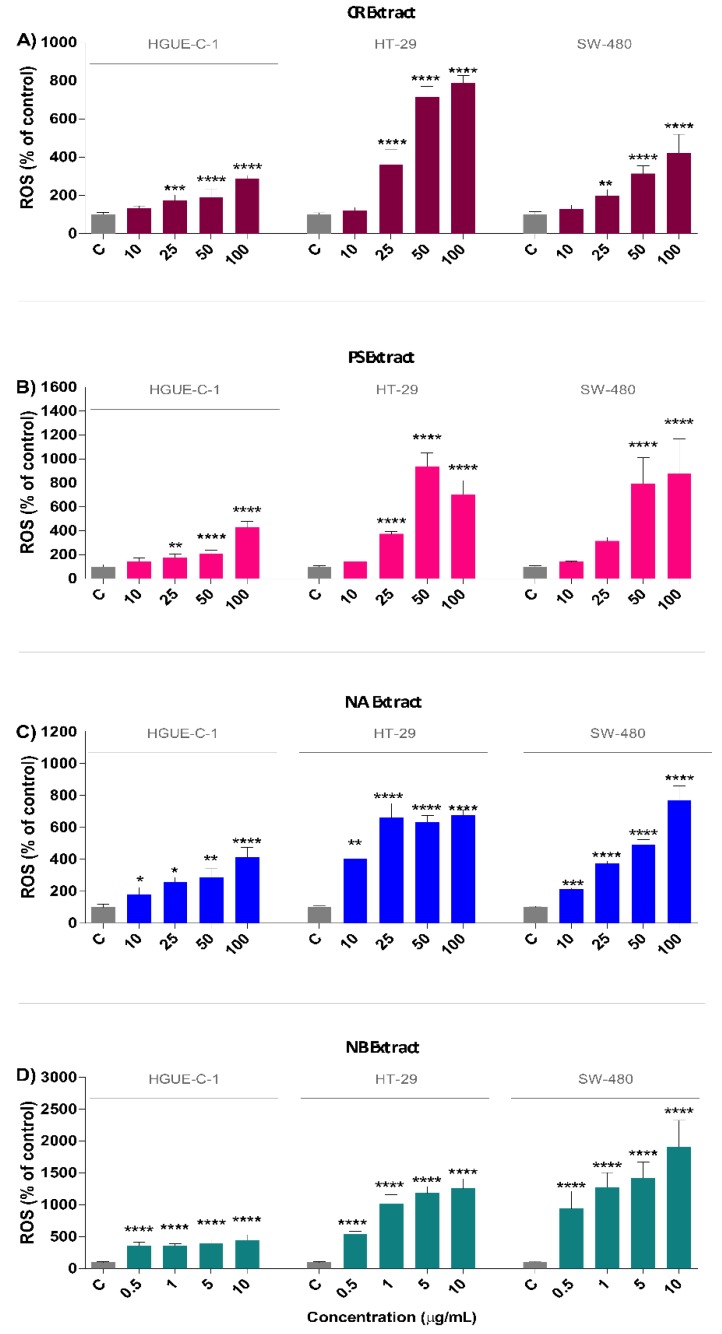
Treatment with marine extracts causes intracellular ROS generation in human colon carcinoma cells. Intracellular ROS levels were assessed by measuring the intensity of dichlorodihydrofluorescein diacetate (H_2_DCF-DA) fluorescence in HGUE-C-1, HT-29, and SW-480 cells after 24 h of treatment with the CR extract (**A**), PS extract (**B**), NA extract (**C**), and NB extract (**D**) and comparing the intensities to those of the respective untreated cells (control cells administered less than 0.2% dimethyl sulfoxide, C). The results are presented as the percentages (means ± SD) from three independent experiments. *p*-values were calculated and compared to the same untreated cell line using ANOVAs. * *p*-value < 0.05, ** *p*-value < 0.01, *** *p*-value < 0.001, and **** *p*-value < 0.0001.

Due to their pleiotropic role in a large variety of cellular processes, ROS may function either as mediators or triggers of the apoptotic or necrotic cell death pathways in cancer cells. We therefore sought to determine whether the marine extracts affected cellular ROS accumulation. For this purpose, cells were exposed to different concentrations of marine extracts for 24 h and then stained with 2,7-dihydrodichlorofluorescein diacetate (H_2_DCF-DA) to detect intracellular ROS levels. ROS levels in HGUE-C-1, HT-29, and SW-480 cells were consistently and significantly increased in response to treatment with the marine extracts in a dose-dependent manner compared to the levels observed in untreated cells (Figure 6). Among the tested marine extracts, the NB extract exhibited the most potent effect on intracellular ROS accumulation, followed by PS, NA, and CR. NB increased the ROS levels by 4.5-fold, 12.6-fold, and 10.1-fold in HGUE-C-1, HT-29, and SW-480 cells, respectively, compared to the levels in the control cells at the maximum concentration tested, i.e., 10 µg/mL (Figure 6D). PS increased ROS accumulation 4.3-fold, 7.0-fold, and 8.9-fold in HGUE-C-1, HT-29 and SW-480 cells, respectively, at 100 µg/mL (Figure 6B). The NA extract at 100 µg/mL increased ROS levels 4.2-fold, 6.8-fold, and 7.7-fold in HGUE-C-1, HT-29, and SW-480 cells, respectively, compared to the levels in untreated cells (Figure 6C). Finally, the CR extract at 100 µg/mL increased ROS levels 2.9-fold, 7.9-fold, and 4.2-fold in HGUE-C-1, HT-29, and SW-480 cells, respectively, compared to levels in the untreated cells (Figure 6A).

**Figure 7:**
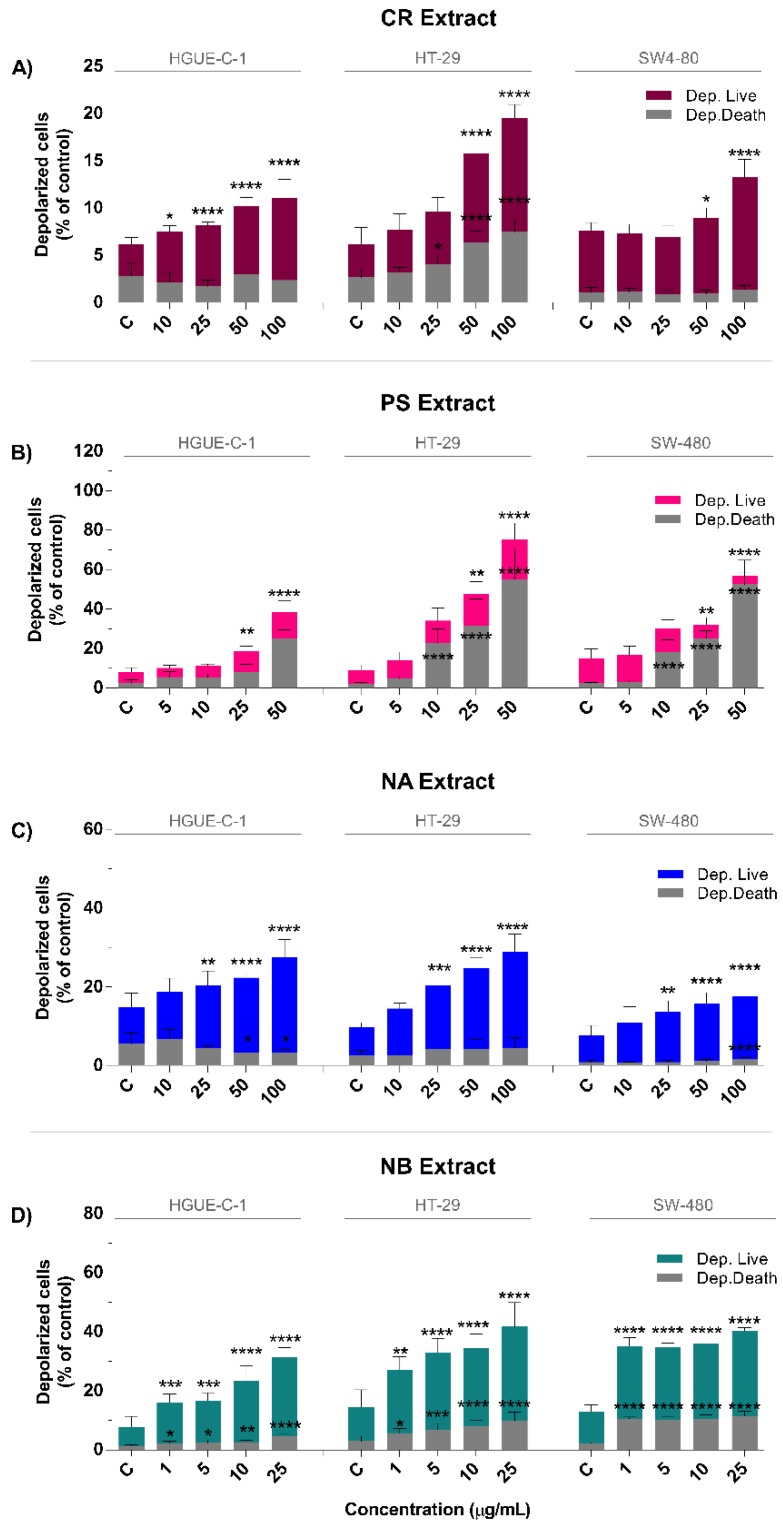
Marine extracts cause mitochondrial membrane depolarization in human colon carcinoma cells. HGUE-C-1, HT-29, and SW-480 cells were treated with different concentrations of CR extract (**A**), PS extract (**B**), NA extract (**C**), and NB extract (**D**) for 24 h and harvested as indicated in the Materials and Methods section. Depolarized live (Dep. Live) cells and depolarized dead (Dep. Dead) cells were measured using the Muse^®^ Cell Analyzer. The results are presented as the percentages (means ± SD) from three independent experiments. *p*-values were calculated and compared to the same untreated cell line (control cells plus dimethyl sulfoxide less than 0.2%, C) using ANOVAs. * *p*-value < 0.05, ** *p*-value < 0.01, *** *p*-value < 0.001, and **** *p*-value < 0.0001.

Changes in the mitochondrial membrane potential (MMP) that compromise mitochondrial function are related to metabolic stress triggered by ROS generation and inflammation. Moreover, variations in the MMP are also an indicator of irreversible checkpoints in the apoptotic process [23]. We analyzed the MMP using the cell analyzer Muse^®^, which allows us to measure the changes in the mitochondrial membrane potential using a cationic, lipophilic dye and in cellular plasma membrane permeabilization or cell death using 7-AAD. The effects of the different concentrations of marine extracts on mitochondrial membrane depolarization (loss of ∆ψm) were measured in HGUE-C-1, HT-29, and SW-480 cells exposed to the marine extracts for 24 h. Figure 7A–D shows the effects of the marine extracts on the depolarization of mitochondrial membranes, with depolarized live (Dep.Live) cells and depolarized dead (Dep.Dead) cells. High ratios of Dep.Live to Dep.Dead cells may suggest an apoptotic-mediated mechanism, whereas lower ratios indicate a necrotic process; thus, this metric may be an indicator of the type of cell death caused by marine extracts. In general, compared to no treatment, CR, PS, NA, and NB all increased the proportions of depolarized cells in a dose-dependent manner (Figure 7). The results showed dissimilar behaviors of the NB, CR and NA marine extracts compared to those of the PS extract. The NB, CR, and NA extracts increased the proportion of Dep.Live cells compared to the Dep.Dead cell population, which is characteristic of an apoptotic process, whereas PS increased the proportion of Dep.Dead cells compared to Dep.Live cells, which is typical of a nonapoptotic process.

Treatment with 100 µg/mL of the CR extract increased the percentage of Dep.Live cells from 3.4 ± 0.8% in control cells to 8.7 ± 2.0% in HGUE-C-1 cells, from 3.4 ± 1.8% to 12.0 ± 1.5% in HT-29 cells, and from 6.5 ± 0.8% to 11.8 ± 2.0% in SW-480 cells (Figure 7A). The NA extract increased the percentage of Dep.Live cells to a greater extent than the percentage of Dep.Dead cells in a dose-dependent manner, producing an effect that was 2–15 times stronger depending on the cell line and the extract concentration. Upon treatment with 100 µg/mL NA, the percentage of Dep.Live cells increased from 9.4 ± 3.5% in control cells to 24.3 ± 4.4% in treated HGUE-C-1 cells, from 7.1 ± 1.2% to 24.4 ± 4.5% in treated HT-29 cells, and from 6.8 ± 2.6% to 16.1 ± 3.9% in SW-480 cells. NA also induced a significant but weaker increase in the percentage of Dep.Dead SW-480 cells at high concentrations (Figure 7C). The NB extract increased the percentages of both types of depolarized cells in a dose-dependent manner, but the increase in the percentage of Dep.Live cells was more pronounced, i.e., two-fold to five-fold higher, than that of Dep.Dead cells. Treatment with 25 µg/mL NB increased the percentage of Dep.Live cells from 6.4 ± 3.6% in control cells to 26.9 ± 3.1% in treated HGUE-C-1 cells, from 11.4 ± 6.0% to 31.7 ± 8.2% in treated HT-29 cells, and from 10.8 ± 2.2% to 28.8 ± 1.0% in treated SW-480 cells (Figure 7D). PS promoted a significant decrease in the percentage of Dep.Live cells in some cell lines and increased the proportion of Dep.Dead cells from two-fold to five-fold compared to control cells. At 100 µg/mL, PS increased the percentage of Dep.Live cells from 5.4 ± 2.2% to 13.2 ± 5.7% in HGUE-C-1 cells, from 6.7 ± 2.8% to 20.2 ± 8.0% in HT-29 cells, and from 12.6 ± 5.0% to 20.2 ± 8.0% in SW480 cells. However, a higher percentage of Dep.Dead cells than Dep.Live cells was observed in most cases, indicating a marked difference in the death mechanism. The data revealed that 100 µg/mL PA induced substantial changes in the percentage of Dep.Dead cells from 2.7± 1.4% in the control to 25.3 ± 4.2% in HGUE-C-1 cells, from 2.2 ± 0.4% to 55.3 ± 15.4% in HT-29 cells and from 2.3 ± 0.4% to 52.7 ± 12.2% in SW-480 cells (Figure 7B).

These effects on the MMP were confirmed using alternative techniques, such as the measurement of the ratio between MitoTracker Red (MitoRed), which identifies viable mitochondria, and MitoTracker Green (MitoGreen), which labels all mitochondria, as described in the Supplementary Information and Supplementary Figures S7 and S8.

### 2.5. Marine Extracts Induce Cell Death by Activating Caspases

**Figure 8:**
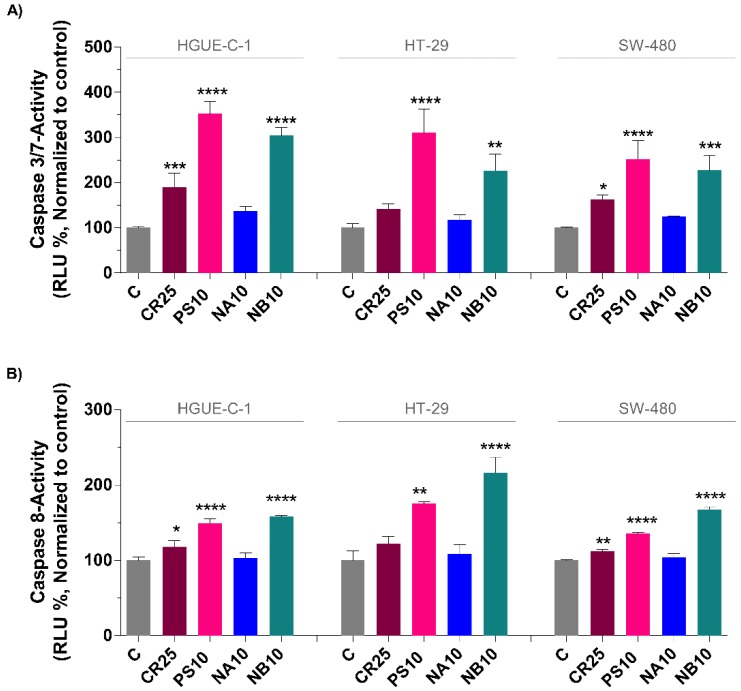
Induction of apoptosis in colon cancer cells by the CR, PS, NA, and NB marine extracts via activation of caspases. The activities of caspases 3/7 and 8 in colon cancer cells treated with the extracts (CR at 25 µg/mL, PS at 10 µg/mL, NA at 10 µg/mL and NB at 10 µg/mL) for 24 h were determined using the Caspase-Glo 3/7 (**A**) and Caspase-Glo 8 (**B**) assays. The results were compared to the respective untreated cells (control cells administered less than 0.2% dimethyl sulfoxide, C) and presented as the percentages (means ± SD) from three independent experiments. *p*-values were calculated and compared to the corresponding untreated cell line using ANOVA. * *p*-value < 0.05, ** *p*-value < 0.01, *** *p*-value < 0.001, and **** *p*-value < 0.0001.

Caspases are highly specific proteases that are activated by cleavage in the apoptosis pathway and are responsible for initiating the death of mammalian cells. However, it is known that caspases are also involved in nonapoptotic pathways [24,25]. To determine the effect of the marine extracts on inducing apoptosis and to confirm our previous results from the Annexin V and MitoPotential assays, the activation of executioner caspases 3/7 and the initiator of the extrinsic apoptosis caspase 8 [26,27] was measured in colon cancer cells treated with different marine extracts for 24 h. The results showed that CR, PS, and NB were able to significantly increase the activity of caspases 3/7 and 8 compared to that in control cells (Figure 8). However, the NA extract was not able to induce a noticeable activation of caspases 3/7 and 8.

The PS extract induced the most substantial increase in caspase 3/7 activity compared to the other extracts (Figure 8A). Treatment with the PS extract (10 µg/mL) increased caspase 3/7 activity 3.5-fold in HGUE-C-1 cells, 3-fold in HT-29 cells, and 2.5-fold in SW-480 cells. NB also significantly increased caspase activation 3-fold in HGUE-C-1 cells and up to 2.3-fold in HT-29 and SW-480 cells compared to the activation in the respective control cells. CR was less active than PS and NB; however, a significant 2-fold increase in HGUE-C-1 cells and a 1.6-fold increase in SW-480 cells were observed when cells were treated with 25 µg/mL CR.

Among all the marine extracts used, the NB extract exhibited the greatest ability to activate caspase 8, although CR and PS also activated this enzyme. Compared to the control treatment, NB increased caspase 8 activity by approximately 1.6-fold in HGUE-C1 cells, approximately 2-fold in HT-29 cells and 1.7-fold in SW-480 cells. PS increased caspase 8 activation by 1.5-fold in HGUE-C-1 cells, 1.8-fold in HT-29 cells, and 1.4-fold in SW-480 cells. CR again showed the weakest activation, increasing caspase 9 activation by approximately 1.2-fold in all three cancer cell lines (Figure 8B). Finally, similar to its activation of caspase 3/7, the NA extract had no effect on caspase 8 activity.

### 2.6. DNA Damage

An imbalance in ROS triggers and alters a large variety of intracellular processes related to cell stress and the metabolic response, such as the MMP, mitochondrial function, endoplasmic reticulum stress, and the unfolded protein response, histone H2A.X activation, and DNA damage. The putative activation of histone H2A.X by the marine extracts was assessed to investigate their effects on DNA damage. H2A.X is a histone variant of the H2A protein family that forms an octamer in nucleosomes. Factors that cause DNA double-strand breaks (DSBs) activate ataxia telangiectasia mutated (ATM) kinase through phosphorylation. Activated ATM is one of the mediators of histone H2A.X phosphorylation on Ser139, which leads to the formation of γ-H2AX; this protein is recruited to DSBs and initiates local DNA repair [28].

**Figure 9:**
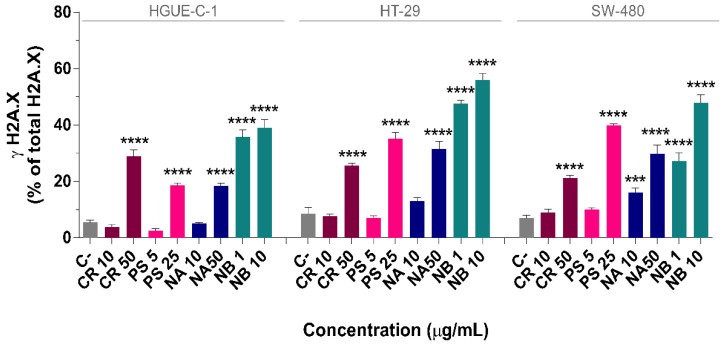
Marine extracts induce histone H2A.X phosphorylation, an indicator of DNA damage, in colon cancer cells. The extent of DNA damage produced by marine extracts was assessed by measuring the levels of phosphorylated H2A.X histone (γ-H2AX) (% of γ-H2AX to total H2A.X) in HGUE-C-1, HT-29, and SW-480 cells after treatment with 10 and 50 µg/mL CR or NA, 5 and 25 µg/mL PS, and 1 and 10 µg/mL NB for 24 h. The results were compared to those of untreated cells (control cells administered less than 0.2% dimethyl sulfoxide, C) and are presented as the percentages (means ± SD) from three independent experiments. *p*-values were calculated and compared to the same untreated cell line using ANOVAs. *** *p*-value < 0.001, and **** *p*-value < 0.0001.

Treatments with marine extracts produced high levels of the active form of γ-H2AX in most cases (Figure 9), suggesting that the increase in ROS levels described above is related to this response and results in significant DNA damage. Among the four marine extracts, NB promoted a substantial increase in H2A.X activation both at the lowest (1 µg/mL) and highest concentrations (10 µg/mL) tested, indicating extensive DNA damage. At 10 µg/mL, NB increased the level of γ-H2AX from 5.5 ± 0.8% in control cells to 39.0 ± 2.9% in HGUE-C-1 cells, from 8.6 ± 2.2% to 56.1 ± 2.3% in HT-29 cells, and from 6.9 ± 1.1% to 47.9 ± 2.9% in SW-480 cells.

The CR, PS, and NA extracts also significantly increased the phosphorylation of H2A.X at the highest concentrations tested. Treatment with 50 µg/mL of the CR extract increased H2A.X phosphorylation from 5.5 ± 0.8% in control cells to 28.9 ± 2.2% in HGUE-C-1 cells, from 8.6 ± 2.2% to 25.6 ± 0.9% in HT-29 cells and from 6.9 ± 1.1% to 21.3 ± 0.9% in SW-480 cells. Likewise, 25 µg/mL PS enhanced H2A.X activation from 5.5 ± 0.8% in control cells to 18.7 ± 0.8% in HGUE-C-1 cells, from 8.6 ± 2.2% to 35.1 ± 2.4% in HT-20 cells, and from 6.9 ± 1.1% to 40.0 ± 0.6% in SW-480 cells. Finally, 50 µg/mL NA increased γ-H2AX levels from 5.5 ± 0.8% in control cells to 18.5 ± 0.9% in HGUE-C-1 cells, from 8.6 ± 2.2% to 31.5 ± 2.8% in HT-29 cells, and from 6.9 ± 1.1% to 29.9 ± 3.0% in SW-480 cells.

### 2.7. Characterization of Marine Invertebrate Extracts Using HPLC-ESI-TOF-MS

Among marine invertebrates, substantial intraspecies variability exists in the production of secondary metabolites; this phenomenon not been well defined [29]. This study is one of the most comprehensive investigations of the characterization of secondary metabolites from different marine sources (two nudibranchs, one holothurian and one soft coral) using HPLC coupled to mass spectrometry to date.

The extracts of the four selected marine invertebrates were analytically characterized using HPLC-ESI-TOF-MS in both positive and negative modes, as described in the Materials and Methods section. Their base peak chromatograms (BPCs) are depicted in Supplementary Figures S9 and S10, and peaks were identified using the approach described in the Materials and Methods section. This nontargeted approach allowed us to identify 21 compounds in CR, 24 compounds in PS, 25 compounds in NA and 31 compounds in NB. These compounds belong to different chemical classes, i.e., terpenes, peptides, fatty acids, alkaloids, and polyketides, among others, and accounted for 98 different metabolites.

Table 3, Table 4, Table 5 and Table 6 show the retention time (RT), experimental *m*/*z* of both negative ([M−H]^−^) and positive ([M−H]^+^) molecular ions, molecular formula, mass error, normalized area, and the proposed identification of each compound. Compounds were numbered according to their elution order. Compounds reported for the first time in any marine organism investigated in the present study are marked with an asterisk (*). These tables also include the bibliographic references reporting the antiproliferative or anticancer activities of these compounds. Further data used for identifying peaks are extensively described in the Supplementary Information and addressed in the Discussion section.

## 3. Discussion

Anticancer research and the discovery of new drugs are continuously increasing the therapeutic arsenal available to face oncological challenges. CRC is the third most prevalent cancer worldwide, and despite the great advances obtained in both treatment options and survival rates, new therapeutic approaches are still needed, particularly for patients with the worst prognosis. Therefore, efforts aimed at identifying new anticancer drugs, not only in the context of CRC but also for other types of cancer, are always worthwhile. Natural compounds have been traditionally used as an almost limitless source of new molecules that have been selected through evolution and, in many cases, have exerted a hermetic effect and multitargeted activity in humans [3]. Among the natural compounds discovered, products from marine organisms have emerged more recently [10]. Marine organisms survive in a harsh microenvironmental ecosystem, leading to the generation of a sophisticated arsenal of metabolites with greater chemical novelty and diversity than terrestrial metabolites [10]. Thus, marine organisms, particularly marine invertebrates, are an excellent resource for the discovery of anticancer compounds [10,113].

Among the 20 marine invertebrates screened in the present study for their antiproliferative activities toward a panel of human colon cancer cell lines (Table 1, Supplementary Table S2; Table 3 and Supplementary Figure S1), four extracts from *Carotalcyon* sp. (CR, soft coral), *Pseudocolochirus violaceus* (PS, holothurian), *Phyllidia varicosa* (NA), and *Dolabella auricularia* (NB), both of which are nudibranchs, were selected based on their strong antiproliferative effect.

The four selected extracts decreased cell viability at fairly low doses, in contrast to some previous reports [114,115]. This antiproliferative effect was observed rapidly after cells were treated (particularly with the PS and NB extracts), as evidenced by morphological alterations such as cell shrinkage, membrane blebbing and rounded or detached cells (which were observed on a Cell Imaging Multi-Mode Reader). The antiproliferative potential of the CR, PS, NA, and NB extracts was further confirmed by their ability to decrease the number of viable cells, and the proliferation rates were monitored via RTCA to evaluate the inhibition of the capacity of the colon cancer cells to generate new colonies in the clonogenic assay ( Figure 1; Figure 2).

The present study revealed a potent induction of G2/M arrest together with a concomitant decrease in the proportion of cells in S phase by the extracts in a dose-dependent manner. One of the factors that could contribute to the cell cycle arrest of colon cancer cells at G2/M phase is the phosphorylation of H2A.X, which was observed in all cell lines treated with any of the extracts (NB in particular). H2A.X phosphorylation indicates the presence of DSBs and DNA damage (Figure 9). Cell cycle checkpoints are essential to maintain the genomic integrity of proliferating cells. In response to DNA damage, cells must detect and repair the damage and either transiently block cell cycle progression to allow time for repair or exit the cell cycle. The reversion of a DNA damage–induced checkpoint requires the repair of these lesions, the prevention of a permanent exit from the cell cycle, and the termination of checkpoint signalling to allow the cell cycle to resume. The severity of the arrest and its reversal depend on the extent of the damage and the cellular repair capacity [116]. Our results show that the NB extract induced particularly extensive DNA damage (although the CR, PS and NA extracts also induced significant damage at high concentrations). This finding might explain the substantial increase in the percentage of cells arrested in G2/M phase, particularly after treatment with NA, NB, and, to a lesser extent, CR. The arrested cells subsequently underwent early apoptosis and probably senescence due to their inability to properly repair damaged DNA (Figure 3 and Figure 4). Compounds that inhibit the abnormal growth of cancer cells by blocking the DNA repair process or functioning as antimicrotubule agents are useful cancer treatments [117,118]. Many compounds isolated from marine sponges, including soblidotin and spongistatins, exhibit potent cytotoxic activities toward several cancer cell lines; these activities are associated with the abilities of these compounds to disturb microtubule homeostasis and to evade the DNA repair system [119,120,121]. 

ROS are pleiotropic and highly reactive molecules that are derived from incomplete oxidative phosphorylation during catabolism but are also produced by exogenous factors such as drugs, UV radiation, and pollutants. The equilibrium of intracellular ROS levels plays an important role in regulating normal physiological cell functions, such as cell cycle progression, proliferation, differentiation, migration, and the activation of different cellular signalling pathways. When the ROS equilibrium is perturbed, these molecules cause severe damage to macromolecules, leading to cell death [122,123].

Interestingly, the four marine extracts selected in the present study induced intracellular ROS production (Figure 6) concomitant with cell cycle arrest and DNA damage accumulation. We postulate that this massive ROS accumulation is responsible for the generation of DNA DSBs and the subsequent activation of the DNA repair machinery, resulting in cell cycle arrest. In most cases, cell repair mechanisms are not sufficient to repair the breaks, and the cell cycle does not resume, which leads the cell to undergo senescence and cell death. The key factors and events that regulate the cellular decision to activate a specific cell death mechanism remain unknown and require further research.

As mentioned above, the NB, NA, and CR extracts significantly increased the percentages of early apoptotic and depolarized live cells. These increases were strongly correlated with membrane blebbing, cellular shrinkage, pyknosis, caspase activation and DNA fragmentation, which are typical features of apoptosis (Figure 4, Figure 5, and Figure 8) [124]. This process was substantially accelerated in HGUE-C-1 cells, which are derived from a primary culture of colorectal adenocarcinoma cells and express wild-type p53, in contrast to the other two cell lines, which express the mutant p53 protein. This difference may explain why these cells exhibited higher apoptosis rates in the cell cycle analysis after treatment with the extracts (Figure 3), similar to the effects of other natural extracts [20]. By contrast, the PS extract showed a less prominent arrest in G2/M phase and substantial increases in the proportions of late apoptotic and depolarized dead cells (Figure 3 and Figure 4). In addition, PS rapidly induced membrane permeabilization (PI/Hoechst staining) and LDH release, which are typical features of the necrosis pathway, in which membrane rupture and rapid lysis of cells and organelles occurs (Figure 5) [125].

In the present study, the different marine extracts appeared to induce cell death through different and complex mechanisms that may overlap to some extent. Our results show increased activation of caspase 3/7 and 8 cleavage by all the extracts except NA. The maximum activation of caspase 3/7 was observed in cells treated with the PS and NB extracts, and the maximum activation of caspase 8 was observed in cells treated with the NB extract. The extrinsic apoptosis signaling pathway is usually activated by the binding of cell death ligands and results in caspase 8-mediated cell death with the subsequent activation of caspases 3/7. By contrast, the intrinsic pathway (mitochondrial or BCL-2-regulated pathway) is activated by cellular stress and chemotherapeutics and includes proapoptotic BCL-2 family protein activation, Cytochrome C release, and the subsequent activation of caspases 3/7.

Because PS, which appeared to promote necrosis as the predominant death mechanism (as determined by LDH activity and fluorescence microscopy), also activated caspase 8 and caspase 3/7, we propose that the main cell death mechanism activated by this extract might be the extrinsic pathway. This option would also be compatible with a death mechanism that is less dependent on mitochondrial depolarization and promptly ends in necrosis. Moreover, the increase in caspase activity in necrotic processes has been previously described in other studies [126,127] and was linked to the presence of ROS, as observed in the present study. Furthermore, at least for the PS extract, our results might indicate the activation of both apoptosis and necrosis to induce cell death; one pathway would predominate, depending on each single case. Since the marine extracts are very complex mixtures, a reasonable hypothesis is that the different compounds in each extract simultaneously induce different death mechanisms [128]. Furthermore, even when a single death mechanism prevails, crosstalk with another cell death mechanism may occur [129,130].

In addition, cancer cell lines, which are usually fairly resistant to cell death, are highly diverse and heterogeneous and may present substantial alterations in their proliferation pathways and mechanisms that orchestrate cell death. Thus, the typical characteristics of a specific cell death process, such as apoptotic or nonapoptotic cell death, may vary appreciably [131,132,133]. Moreover, important events related to the control of cell cycle checkpoints or even defective apoptosis may result in cell death mechanisms, as evidenced in programmed cell death. In this context, mitotic catastrophe (delayed mitosis-linked cell death), which occurs in cells experiencing cell cycle arrest and a lack of doubling, shares characteristics with apoptosis and necrosis and manifests features typical of both processes [134].Taken together, these results describe a complex scenario that should be revisited in future studies.

Current anticancer treatments are accompanied by several undesired consequences, such as toxic effects. Therefore, new anticancer drugs must exert therapeutic effects and induce cell death in a controlled manner [135]. Cytotoxic compounds have been shown to induce cell death via necrosis, autophagy or apoptosis, which is desirable for a new candidate anticancer agent [136], but further studies must be pursued in the future to validate whether the extracts used in this work present an adequate therapeutic index.

This study also elucidated the chemical composition of the CR, PS, NA, and NB extracts using HPLC-ESI-TOF-MS and revealed extremely diverse and complex compositions. The chemical characterization revealed a wide variety of metabolites that were mainly classified as terpenes, peptides, fatty acids, alkaloids and polyketides, among others; in all, 98 compounds were identified. The majority of the identified compounds were previously reported in the marine ecosystem; meanwhile, others were identified in terrestrial habitats. However, some of the compounds identified were found in a marine source for the first time. Some of the compounds identified in these marine extracts have previously been reported to inhibit a few hallmarks of cancer in vivo*,* as discussed below.

The antiproliferative activities of up to 75 of the 98 identified compounds have been reported in the literature, accounting for 75.8% of the total identified compounds. Nevertheless, the semiquantitative analysis (as determined by the larger normalized area percentage) of the compounds present in the marine extracts utilized revealed that the observed antiproliferative effects might be associated with particular families of compounds (Table 3, Table 4, Table 5 and Table 6). For instance, the main candidates to account for the antiproliferative effects of the CR extract might be diterpenes such as spongian-16-one [40], polyoxygenated marine steroids such as punicinol D [52], sesquiterpenes such as dendronephthol C [37], or sesterterpenes such as deoxoscalarin [38]. In the PS extract, furanocembranolide lopholide [56,64] seems to be the most abundant compound with a reported antiproliferative capacity in cancer cells. 

Major compounds in the NA extract were long fatty acids and lyso-PAF, but other compounds with previously reported antiproliferative activity, such as the diterpene spongian-16-one [40], the chlorophyll ethyl pheophorbide A [88,112], palmerolide A (a macrocyclic polyketide) [85,86], and rhizovarin D (an indole diterpene) [89], were present in large amounts. Finally, the most abundant compounds in the NB extract were spongian-16-one and a porphyrin derivative, pyropheophorbide A, both of which have been reported to possess antiproliferative activity [40,88,112]. Moreover, purpurogemutantin (a drimenyl cyclohexenone derivative) [110], trofoside A (polar steroids) [108], and aplysioviolin (a tetrapyrrolic chemodeterrent ink) [103,104] were also fairly abundant in this extract. In any case, studies focused on the fractionation of these extracts may deserve further attention to identify the most active compounds/fractions to be utilized in animal trials.

**Figure 10:**
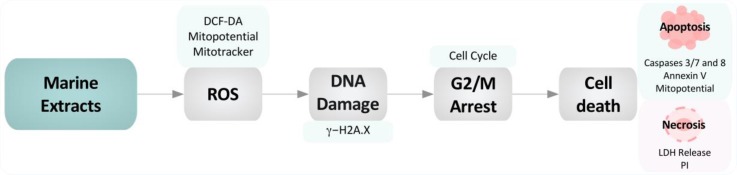
Model depicting the putative mechanisms of action of invertebrate marine extracts in colon cancer cell models. Marine extracts trigger the intracellular accumulation of reactive oxygen species (ROS) that lead to decreased mitochondrial membrane potential (MMP) and mitochondria depolarization. Due to the presence of ROS, H2A.X is phosphorylated as a warning signal for DNA damage, leading to subsequent G2/M arrest. Afterward, if the metabolic stress exceeds the cell stress response, cells undergo either apoptotic or necrotic cell death, depending on the type of marine extract and the extent of the damage.

In conclusion, the four invertebrate marine extracts selected and studied in this investigation may become suitable agents for the further development of anticancer agents against CRC. All the extracts studied here showed a strong antiproliferative capacity and induced G2/M cell cycle arrest that evolved to early apoptosis in the case of the CR, NA, and NB extracts. However, PS exerted its antiproliferative effects by inducing necrotic cell death or a combination of necrosis and the extrinsic apoptotic pathway. We propose that intracellular ROS accumulation triggered by the extracts is responsible for the subsequent DNA damage, mitochondrial depolarization and cell cycle arrest, all of which ultimately induce cell death either by an apoptotic or necrotic mechanism, depending on the extract (Figure 10). Further studies focused on extract fractionation, isolation of the compounds responsible for the observed effects, and obtaining additional information about the putative molecular mechanism may provide new insights into the development of new drug treatments for colon cancer.

## 4. Materials and Methods

### 4.1. Chemicals and Reagents

Organic solvents including dichloromethane, methanol, n-butanol, ethyl acetate, n-hexane were the reagents used for extracting the bioactive compounds from marine organisms, and HPLC-grade acetonitrile, acetic acid and dimethyl sulphoxide (DMSO) were purchased from Sigma-Aldrich (Darmstadt, Germany). Water was purified using a Milli-Q system (Millipore, Bedford, MA, USA). Labels and reagents such as Thiazolyl Blue Tetrazolium Bromide (MTT), Hoechst 33342 propidium iodide (PI), and 2′,7′-Dichlorodihydrofluorescein diacetate (H_2_DCF-DA) were purchased from Sigma-Aldrich. 

### 4.2. Marine Invertebrate Material

Marine invertebrates were selected based on observations of inter and intra-specific competition in experimental aquariums and searches on bibliographic bases [10]. They were obtained from the distributor company of marine species TodoPez S.L. (Alicante, Spain). Twenty species of marine invertebrates were chosen for their potential as producers of compounds with anticancer activity. The selected species were composed of 12 soft corals, four hard corals, two nudibranchs, one anemone, and one holothurian. For more details, see Table 1. 

### 4.3. Extraction Method and Preparation of Crude Extracts

The freshly marine invertebrates were set free of any debris, cut into small pieces, and weighed. The mass was macerated with dichloromethane:methanol (D:M) (1:1, *v*:*v*) at 4 °C for 24 h. After maceration, the solution was filtered and evaporated to dryness on a rotatory vacuum evaporator (Büchi Labortechnik AG, Flawil, Switzerland). The extracts were dried by speed vac (Genevac™ miVac Centrifugal Concentrators, Ipswich, UK), weighted and stored at −80 °C until used. The yield of solid extraction ranged from 1 to 11%, depending of the type of organism (Supplementary Table S1). For *in vitro* culture, extracts were dissolved in DMSO (50 mg/mL) and further diluted in culture medium yielding a final testing concentration of 100 μg/mL with a final DMSO concentration of 0.2%. This concentration of DMSO did not affect cell viability. This methodology was extracted from from [113].

### 4.4. Cell Culture

The human colorectal carcinoma cell lines HT-29 (catalogue number HTB-38) and SW-480 (catalogue number CCL-228) were purchase from the American Type Culture Collection (ATCC, Wesel, Germany) and HGUE-C-1 (an established cell line from primary human colon carcinoma) provided by Dr. Miguel Saceda (Hospital General Universitario de Elche, Elche, Spain). In colorectal cancer disease, there are heterogeneous markers that have important implications for prognosis and clinical treatments. Some of the widely studied markers are the microsatellite instability (MSI), which supposes genetic hypermutability that results from impaired DNA mismatch repair in repetitive DNA sequences called microsatellites, and somatic mutations in BRAF and KRAS, which represent a potential molecular marker for the risk of developing advanced neoplasia [137]. The cell lines used were chosen because of their capability to present some of these markers. The cell line HGUE-C-1 was produced by a primary culture in 2003, derived from ascitic efussion of a 76-year-old patient with colon cancer at the University General Hospital of Elche. This line supposes an interesting model for the study of chemoresistance due to is characterized by presenting resistance in vivo to the drugs 5-fluorouracil and Irinotecan. HGUE-C-1 is stable for microsatellite (MS) phenotype and showed wild type to KRAS and BRAF mutations [138]. HT-29 is an established cell line isolated from a primary tumor in 1964 from a 44-year-old caucasian female. HT-29 showed a phenotype of human colon adenocarcinoma moderately differentiated to grade II. This line presents epithelial morphology, is stable for MS, presents a wild type phenotype to KRAS and is mutated in BRAF (V600E) [139]. The SW480 cell line was isolated from a primary colon tumor of a 50-year-old Caucasian man. It presents epithelial morphology and mutation in the oncogene KRAS (G12V), is wild type to BRAF, and is stable for MS [140].

All cell lines were maintained in Dulbecco’s modified Eagle’s medium (DMEM), supplemented with a 10% heat inactivated fetal bovine serum (FBS), 100 U/mL penicillin, and 100 g/mL streptomycin. Cells were incubated at 37 °C in a humidified atmosphere containing 5%/95% of CO_2_/air. Cells were trypsinized with 0.05X trypsin/ethylene diamine tetra-acetic acid every three days according to the manufacturer’s instructions. Cell culture reagents were purchased from Invitrogen Life Technologies (Carlsbad, CA, USA).

### 4.5. MTT Cell Viability Assay and IC50 Determination

HGUE-C-1, HT-29, and SW-480 cells were plated in 96-multiwell culture plates at a density of 5 × 103 cells/well. Cells were counted using a CytoSmart cell counter (CytoSMART Technologies BV, Eindhoven, the Netherlands). After 24, 48, and 72 h incubation with the crude extracts, MTT was added at a final concentration of 0.5 mg/mL and incubated for 3 h. Then, the medium was removed, and the formazan crystals were dissolved in DMSO. Absorbance was measured at 570 nm in a microplate reader (SPECTROstar Omega, BMG Labtech, Offenburg, Germany). The IC50 values were determined using GraphPad Prism v5.0 software (GraphPad Software, La Jolla, CA, USA). The results are expressed as the percentage of cell viability/proliferation relative to the same untreated cell line (control cells plus dimethyl sulfoxide at 0.2%, C).

### 4.6. RTCA Proliferation Assay

The proliferation rate was monitored in real-time using the xCELLigence system E-Plate Real Time Cell Analyzer (RTCA) (Roche Diagnostics GmbH, Mannheim, Germany). This system collects impedance values of each well and translate to a cell index value (CI). Colon cancer cells were seeded at a density from 7.5 × 10^3^ to 2.0 × 10^4^ cells/well depending on the cell line, adjusted for 24 h after the seed, and the CI was near 1 as manufacturer’s recommendations. HGUE-C-1, HT-29, and SW-480 cells were treated with marine extracts at 10, 25, 50, and 100 µg/mL, and CI was automatically monitored for duration of 75 h, which produced a kinetic curve. Data for cell proliferation was normalized at CI from 24 h (when the treatment was added). 

### 4.7. Clonogenic Assay

Colon cancer cells (HGUE-C-1, HT-29, and SW-480) were seeded in six-well plates at a density of 5 × 10^3^ cells per well and treated the day after with different concentrations of marine extracts (CR, PS, NA and NB) for a period of 24 h. After removing the treatment, fresh media was added, and cells were kept in the tissue culture incubator for an additional six days without disturbance to allow colony formation (capacity of cells to produce progeny from a single cell to form a colony). Cell nucleus were stained with Hoechst 33342 and fixed with 70% ethanol for 5 min for the determination of number and diameter of colonies by the Cell Imaging Multi-Mode Reader Cytation 3 (BioTek Instruments, Inc., Swindon, United Kingdom). Then cells were stained with 0.05% crystal violet for 10 min to take photos of plates. 

### 4.8. Cell Cycle Analysis

Here,1.5 × 10^5^ cells per well were seeded into six-well plates and treated with marine extracts at different concentrations for 24 h and then harvested and washed with phosphate buffered saline (PBS) 1×. The washed cells were fixed with 70% ethanol. The different stages of the cell cycle were determined based on differential DNA content using propidium iodide (PI) which is a nuclear DNA intercalating stain. This measure was made according to the manufacturer’s instruction by the Muse^®^ Cell Analyzer (Merck, Darmstadt, Germany).

### 4.9. Measurement of Apoptosis by Annexin V and Mitopotential

Here, 1.5 × 10^5^ cells per well were seeded into six-well plates and treated with marine extracts at different concentrations for 24 h and then harvested and washed with PBS 1X. The apoptosis was determined by using The Muse^®^ Annexin V and Dead Cell kit based on the detection of phosphatidylserine (PS) on the surface of apoptotic cells, using the fluorescent label Annexin V. 

Mitopotential assay utilizes the MitoPotential Dye (a cationic and lipophilic dye) to detect changes in the mitochondrial membrane potential (MMP) and is combined with 7-AAD as an indicator of cell death. Changes in MMP is a hallmark of permeabilization of cellular plasma membrane and the apoptotic process. The measure was made according to the manufacturer’s instruction by the Muse^®^ Cell Analyzer (Merck, Darmstadt, Germany).

### 4.10. Caspase 3/7 and 8 Activation 

For caspase 3/7 and 8 activity determination, HGUE-C-1, HT-29 and SW480 cells were seeded (2 × 10^5^ cells) into a six-well plate. After 24 h of incubation with marine extracts at different concentrations, cells were lysed with cell lysis buffer and the amount of protein were determined by BCA Pierce Method. Caspase-3 and 7 and Caspase-8 were measured using a Caspase-Glo 3/7 and Caspase-Glo 8 assay kit (both from Promega, Madison, WI, USA) following the manufacturer’s instruction. Total cell lysates containing equal amounts of proteins were gently mixed with Caspase-Glo 3/7 and 8 kit (ratio 1:1) and incubated for 30–40 min at room temperature in the dark. Luminiscence was measured using the Cell Imaging Multi-Mode Reader Cytation 3.

### 4.11. Lactate Dehydrogenase (LDH) Measurement for Necrosis Assay

LDH cytotoxicity assay kit (Roche Diagnostics, Mannheim, Germany) was employed to assess the cellular toxicity induced by compounds as a measure of permeabilization of plasma membrane like a key signature for necrotic process [141]. The measurement is based on the reduction of nicotinamide adenine dinucleotide (NADH) by LDH in the oxidation of lactate to pyruvate process, transforming the tetrazolium salt to a coloured formazan product. The amount of formazan product is determined by spectrophotometry and it is proportionally to LDH activity. HT-29, SW-480, and HGUE-C-1 cancer cells were treated with different concentrations of CR, PS, NA, and NB extract for 24 h, then the supernatant was used to assay LDH activity, according to the manufacturer’s instruction (Roche Diagnostic Systems, Montclair, NJ, USA). The concentration of LDH was measured with a microplate reader (SPECTROstar) at a wavelength of 490 nm. 

### 4.12. Nuclear Staining with Hoechst 33342/Propidium Iodide

Colon cancer cells (5 × 10^5^/well) were seeded into 96-well plates treated with different concentrations of marine extracts (CR, PS, NA, and NB) for a period of 24 h. Cells were washed and incubated with Hoechst 33342 at 5 μg/mL and propidium iodide (PI) at 50 μg/mL for 15 min at 37 °C, then were washed once in PBS (1×). Fluorescece intensity was measured and cells were photographed using a Cell Imaging Multi-Mode Reader Cytation 3 equipped with 40 X objective and fluorescent cubes (DAPI λ = 377–447 and TEXAS λ = 586–647). Results presented are means ± S.D. of three independent experiments.

### 4.13. Mitochondrial Membrane Potential (MMP)

Cell were seeded (5 × 10^5^/well) into 96-well plates and exposed with different concentrations of marine extracts. After treatment cells were washed twice in PBS 1X and incubated with 0.5 μg/mL MitoTracker CMXRos and 15 ng/mL MitoTracker Green (Molecular Probes, Eugene, Oregon, USA) at 37 °C for 45 min. Cells were then washed twice in PBS (1×) and analyzed by the fluorescent wavelength value using the Cell Imaging Multi-Mode Reader Cytation 3. Cells were photographed using a Cell Imaging Multi-Mode Reader Cytation (BioTek Instruments, Inc.) equipped with 20× objective and fluorescent cubes (GFP λ = 469–525 nm and TEXAS λ = 586–647 nm). Results presented are means ± S.D. of three independent experiments.

### 4.14. Detection of Reactive Oxygen Species (ROS) 

Cell were seeded (5 × 10^5^/well) into 96-well plates and treated with different concentrations of marine extracts. The intracellular generation of ROS was measured using 2′,7′-Dichlorodihydrofluorescein diacetate (H_2_DCF-DA, Sigma-Aldrich) (10 μM) and was compared with cell viability using DNA staining Hoechst 33342 (Sigma-Aldrich) (10 μg/mL). After treatment with marine extracts for 24 h, cells were incubated with labels for 30 min in the cell incubator. The levels of intracellular ROS and nucleus number was determined by the fluorescent wavelength value using the Cell Imaging Multi-Mode Reader Cytation 3 (BioTek Instruments, Inc.).

### 4.15. Detection of Phosphorylated H2A.X 

Cells were seeded at 1.5 × 10^5^ cells per well into six-well plates and treated with CR, PS, NA, and NB at different concentrations for 24 h and then harvested and washed with PBS 1×. DNA damage was measured using at the same time a phospho-specific anti-phospho-Histone H2A.X (Ser139)-Alexa Fluor^®^555 and an anti-Histone H2A.XPECy5 conjugated antibody to measure total levels of Histone H2A.X according to the manufacturer’s instruction by the Muse^®^ Cell Analyzer (Merck KGaA).

### 4.16. Determination of Secondary Metabolites by HPLC-ESI-TOF-MS

Analyses of bioactive compounds of these marine organisms were carried out using Agilent 1200 series rapid resolution liquid chromatograph (Agilent Technologies, Palo Alto, CA, USA) that was comprised of a binary pump, degasser, and auto sampler. Compounds were separated at room temperature using a Poroshell 120 EC-C18 (3.6 × 100 mm, 2.7 mm) analytical column (Agilent Technologies). The chromatographic separation was carried out following two different methods depending on the ionization mode. The mobile phases consisted of 1% of acetic acid in water (phase A) and acetonitrile (phase B). The flow rate was set at 0.5 mL/min at 25 °C. The spectra in negative ionization mode was acquired over a mass range from *m*/*z* 50 to 1000, and the following multi-step linear gradient was employed: 0 min, 5% B; 5 min, 15% B; 20 min, 30% B; 35 min, 95% B; 40 min, 5% B. In the method carried out in positive ionization mode the mass scan ranged from *m*/*z* 50 to 1500 and the conditions of the solvent multi-step linear gradient were the following: 0 min, 10% B; 5 min, 65% B; 15 min, 100% B; 19 min, 100% B. 

The HPLC system was coupled to a micrOTOF (Bruker Daltonics, Bremen, Germany), an orthogonal-accelerated TOF mass spectrometer, using an electrospray interface (ESI) (model G1607A from Agilent Technologies, Palo Alto, CA) operating in both negative- and positive-ion modes. The effluent from the HPLC column was splitted using a T-type phase separator before being introduced into the mass spectrometer (split ratio = 1:3). The optimum values for the ESI−MS parameters were as follows: capillary voltage, +4.0 kV; drying gas temperature, 200 °C; drying gas flow, 9.0 L/min; and nebulizing gas pressure, 2.0 bars.

Peak identification was performed by the generation of the candidate formula with a mass accuracy limit of 10 ppm. considering RT, and experimental and theoretical masses. The mass score related to the contribution to mass accuracy, isotope abundance and isotope spacing for the generated molecular formula was set at ≥80. The characterization strategy was based on the interpretation of their mass spectra provided by the TOF-MS and the comparison with previously reported information in the literature for marine invertebrates. For the acquisition of chemical structure information, the following databases were consulted: SciFinder Scholar (http://scifinder.cas.org), MassBank (http://massbank.jp), and METLIN Metabolite Database (http://metlin.scripps.edu). Furthermore, a recent published database of marine natural compounds (http://docking.umh.es/chemlib/mnplib) has been employed.

### 4.17. Statistical Analysis

Significant differences between the mean of control versus various doses were tested by one-way analysis of variance (ANOVA), followed by Tukey’s post hoc test for multiple comparisons using GraphPad Prism version 6.0 (GraphPad Software). Statistically, significant differences were assumed at *p* < 0.05 (* *p* < 0.05, ** *p* < 0.01, *** *p* < 0.001, or **** *p* < 0.0001).

## Figures and Tables

**Table 1:** Identification and codification of the marine species assessed.

Class	Scientific Name	Code	Photo	Class	Scientific Name	Code	Photo
Soft Coral	*Parazoanthus* sp.	P	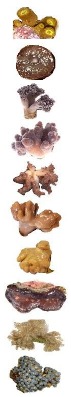	Soft Coral	*Euphyllia ancora*	Eu	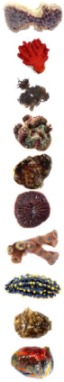
	*Discosoma* sp.	D			*Carotalcyon* sp.	CR	
	*Lemmalia* sp.	L		Anemone	*Aiptasia* sp.	A	
	*Capnella* sp.	C		Hard Coral	*Wellsophyllia* sp.	W	
	*Nepthea* sp.	N			*Echynophyllia* sp.	E	
	*Sarcophyton* sp.	SII			*Fungia* sp.	F	
	*Sinularia* sp.	Si			*Duncanopsamia* sp.	Du	
	*Cataphyllia* sp.	Cy		Nudibranch	*Phyllidia varicosa*	NA	
	*Xenia* sp.	X			*Dolabella auricularia*	NB	
	*Palythoa* sp.	Py		Holothurian	*Pseudocolochirus violaceus*	PS	

**Table 2:** Mean ± SD of the IC_50_ value (μg/mL) of three independent experiments. Values for the other marine extracts are shown in Supplementary Table S2.

	HGUE-C-1			HT-29			SW-480		
Code	24 h	48 h	72 h	24 h	48 h	72 h	24 h	48 h	72 h
**CR**	250.9 ± 92.1	82.0 ± 5.9	58.1 ± 3.4	15.0 ± 4.4	9.4 ± 1.4	10.6 ± 1.0	105.0 ± 10.9	27.6 ± 2.8	14.8 ± 1.6
**PS**	37.5 ± 3. 0	37.4 ± 1.3	48.0 ± 1.8	3.3 ± 1.1	0.7 ± 0.4	2.1 ± 0.7	24.3 ± 2.0	18.6 ± 1.2	16.9 ± 0.6
**NA**	146.0 ± 29.0	61.8 ± 2.9	78.8 ± 3.4	13.0 ± 2.7	10.0 ± 0.7	9.3 ± 1.0	57.2 ± 6.9	13.6 ± 1.5	13.0 ± 2.0
**NB**	11.4 ± 3.4	0.3 ± 0.3	0.1 ± 0.2	5.0 ± 3.6	0.1 ± 0.1	0.1 ± 0.1	54.3 ± 24.2	0.6 ± 0.4	0.2 ± 0.1

**Table 3:** High-performance liquid chromatography coupled to electrospray time-of-flight mass spectrometry (HPLC-ESI-TOF-MS) data of the compounds identified in CR extracts in negative and positive ionization mode. Base peak chromatogram (BPC) is showed in Supplementary Figures S9A and S10A.

**Peak**	**RT ^a^**	***m*/*z* Experimental**	**Molecular Formula (M-H)**	***m*/*z* Calculated**	**Error (ppm)**	**mSigma**	**Identified Compound**	**Area ^b^**	**Identification References**	**Antiproliferative Activity**
1	17.1	171.1017	C_9_H_15_O_3_	171.1027	5.4	29.2	Octenoic acid hydroxy methyl ester isomer 1 *	0.16	[30]	
2	19.12	171.1017	C_9_H_15_O_3_	171.1027	5.4	25.5	Octenoic acid hydroxy methyl ester isomer 2 *	0.08	[30]	
3	25.43	449.1448	C_22_H_25_O_10_	449.1453	1.3	32.9	Asebotin isomer 1 *	0.11	[31]	[31]
4	25.66	153.1277	C_10_H_17_O	153.1285	4.9	62.5	Terpineol *	0.12	[32]	[31]
5	26.13	449.1457	C_22_H_25_O_10_	449.1453	−0.8	36.9	Asebotin isomer 2 *	0.19	[31]	[31]
6	26.65	353.2311	C_20_H_33_O_5_	353.2333	6.3	29.3	Sinulariaoid D	0.05	[33]	[33]
7	26.7	363.2502	C_18_H_31_N_6_O_2_	363.2514	3.4	64.3	Sch 575948 *	0.04	[34]	
8	28.36	439.3304	C_32_H_45_O _4_	439.3323	3.8	39.9	Actinoranone *	0.36	[35]	[35]
9	29.61	255.1588	C_14_H_23_O _4_	255.1602	5.4	87.2	Oxalic acid, allyl nonyl ester *	0.77	[36]	[36]
10	29.66	265.1461	C_15_H_21_O_4_	265.1445	−5.7	24.8	Dendronephthol C	1.73	[37]	[37]
11	33.43	429.2977	C_27_H_41_O_4_	429.3010	7.7	6.5	Deoxoscalarin *	1.65	[37]	[38]
12	36.18	303.2354	C_20_H_31_O_2_	303.2330	−7.9	35.8	Spongian-16-one *	15.00	[39]	[40]
13	37.02	283.2620	C_18_H_35_O_2_	283.2643	8.5	11.7	Stearic acid	3.45	[41,42]	[43]
14	37.18	267.2312	C_17_H_31_ O _2_	267.2330	6.7	3.1	Heptadecenoic acid	6.46	[41,42]	[44]
15	37.7	327.2897	C_20_H_39_O_3_	327.2905	2.4	13	2-Hydroxyeicosanoic acid	4.59	[41,42]	[45]
16	37.84	255.2317	C_16_H _31_O_2_	255.2330	5.3	11.7	Hexadecanoic acid	5.62	[41,42]	[46]
17	38.05	281.2462	C_18_H_33_O_2_	281.2486	8.5	30.8	9-Octadecenoic acid	2.75	[41,42]	[46,47]
18	38.42	357.2772	C_24_H_37_O_2_	357.2799	7.6	8.3	Tetracosapentaenoic acid	6.54	[41,42]	
**Peak**	**RT ^a^**	***m*/*z* experimental**	**Molecular formula (M+H)**	***m*/*z* calculated**	**error (ppm)**	**mSigma**	**Identified compound (positive mode)**	**Area ^b^**	**Identification references**	**Antiproliferative activity**
1	3.6	259.1768	C_15_H_24_NaO_2_	259.1669	−38.5	17.8	Scabralin A	0.50	[48]	[48]
2	8.70	482.3610	C_24_H_53_NO_6_P	482.3605	−1.1	8.1	1-O-hexadecyl-sn-glycero-3-phosphocholine (lyso-PAF) *	27.74	[49]	[50,51]
3	11.43	462.3596	C_28_H_48_NO_4_	462.3578	−3.9	23.4	Punicinol D *	2.35	[52]	[52]

^a^ RT: retention time (minutes). ^b^ Normalized area (%); * Described for the first time in a soft coral.

**Table 4:** HPLC–ESI–TOF–MS data of the compounds identified in PS extracts in negative and positive ionization mode. BPC is showed in Supplementary Figures S9B and S10B.

**Peak**	**RT ^a^**	***m*/*z* Experimental**	**Molecular Formula (M-H)**	***m*/*z* Calculated**	**Error (ppm)**	**mSigma**	**Identified Compound**	**Area ^b^**	**Identification References**	**Antiproliferative Activity**
1	9.81	280.1221	C_15_H_14_N_5_O	280.1204	6.1	42.9	N-[(2E)-3-(2-Amino-1H-imidazol-5-yl)-2-propen-1-yl]-1H-indole-2-carboxamide *	0.11	[53]	[53]
2	14.36	262.1113	C_15_H_12_N_5_	262.1098	−5.8	37.5	Acanthomine A isomer 1 *	0.56	[54]	[55]
3	16.1	262.1105	C_15_H_12_N_5_	262.1098	−2.5	187.6	Acanthomine A isomer 2 *	0.15	[54]	[55]
4	22.36	651.2298	C_31_H_39_O_15_	651.2294	0.3	19	Juncenolide D *	0.61	[56,57]	[58]
5	23.09	290.1416	C_16_H_20_ NO_4_	290.1398	−6.3	19.8	Isopropyl-6-(4-methoxybenzyl)-4-methylmorpholine-2,5-dione *	0.13	[59]	
6	23.43	383.2072	C_20_H_31_O_7_	383.2075	0.8	24.4	Acetyl-methoxydeacetyldihydrobotrydial isomer 1 *	0.04	[60]	
7	23.84	383.2076	C_20_H_31_O_7_	383.2075	−0.1	20.2	Acetyl-methoxydeacetyldihydrobotrydial isomer 2 *	0.04	[60]	
8	23.84	353.1972	C_19_H_29_O_6_	353.1970	−0.7	25.5	Gracilioether A *	0.04	[61,62]	[62,63]
9	24.14	383.2067	C_20_H_31_O_7_	383.2075	2.2	35.7	Acetyl-methoxydeacetyldihydrobotrydial isomer 3 *	0.05	[60]	
10	25.4	455.1522	C_26_H_23_N_4_O_2_S	445.1547	5.6	50.1	Unknown	14.16		
11	25.72	445.1491	C_23_H_25_O_9_	445.1504	3	24	Lopholide *	8.67	[56,57]	[56,64]
12	26.19	621.2349	C_35_H_33_N_4_O_7_	621.2355	1	54.3	Eictenascidin analog *	5.59	[65]	[65]
13	26.4	643.2497	C_25_H_39_N_8_O_10_S	643.2515	2.8	27.1	Unknown	26.67		
14	34.93	301.2162	C_20_H_29_O_2_	301.2173	3.7	1.1	Eicosapentaenoic acid	0.76	[66]	[67,68]
15	36.2	303.2328	C_20_H_31_O _2_	303.2330	0.4	6.6	Arachidonic acid	1.13	[66]	[69]
16	37.04	283.2625	C_18_H_35_O_2_	283.2643	6.2	20.6	Stearic acid	0.82	[66]	[43]
17	37.82	255.2319	C_16_H_31_O_2_	255.2330	4.1	21.8	Hexadecanoic acid *	1.18	[66]	[46]
**Peak**	**RT ^a^**	***m*/*z* experimental**	**Molecular formula (M+H)**	***m*/*z* calculated**	**error (ppm)**	**mSigma**	**Identified compound**	**Area ^b^**	**Identification references**	**Antiproliferative activity**
1	3.96	146.0598	C_9_H_8_NO	146.0600	1.7	15.3	3-Formylindole *	1.61	[70]	[71]
2	6.61	274.2730	C_16_H_36_NO_2_	274.2741	3.7	8.4	2-Amino-1,3-hexadecanediol *	4.87	[72]	
3	7.20	302.3039	C_18_H_40_NO_2_	302.3054	4.9	11.2	Sphinganine 1 *	3	[72]	[73]
4	14.84	597.3906	C_40_H_53_O_4_	597.3938	5.4	29.7	Astaxanthin isomer 1 *	0.5	[74]	[75]
5	16.68	597.3905	C_40_H_53_O_4_	597.3938	5.6	27.1	Astaxanthin isomer 2 *	0.43	[74]	[75]
6	18.64	565.4002	C_40_H_53_O_2_	565.404	6.7	36.7	Canthaxanthin isomer 1 *	0.76	[74]	[76]
7	19.48	565.4040	C_40_H_53_O_2_	565.3999	7.3	12.9	Canthaxanthin isomer 2 *	0.42	[74]	[76]

^a^ RT: retention time (minutes). ^b^ Normalized area (%); * Described for the first time in a sea holothurian.

**Table 5:** HPLC–ESI–TOF–MS data of the compounds identified in NA extracts in negative and positive ionization mode. BPC is showed in Supplementary Figures S9C and S10C.

**Peak**	**RT ^a^**	***m*/*z* Experimental**	**Molecular Formula (M-H)**	***m*/*z* Calculated**	**Error (ppm)**	**mSigma**	**Identified Compound**	**Area ^b^**	**Identification References**	**Antiproliferative Activity**
1	8.03	255.0881	C_12_H_15_O_6_	255.0874	−2.8	22.8	Phenyl β-D-galactopyranoside *	0.21	[77]	[77]
2	12.68	241.0710	C_11_H_13_O_6_	241.0718	3.1	14.3	Tetillapyrone *	0.11	[78]	[79]
3	17.12	167.1067	C_10_H_15_O_2_	167.1078	0.5	17.6	Geranic acid *	0.24	[80]	[81]
4	18.04	420.2317	C_25_H_30_N_3_O_3_	420.2293	−5.9	16.7	Ketopremarineosin A isomer 1 *	1.92	[82]	[83]
5	20.43	420.2299	C_25_H_30_N_3_O_3_	420.2293	−1.5	31.4	Ketopremarineosin A isomer 2 *	1.44	[82]	[83]
6	27.19	353.2312	C_20_H_33_O_5_	353.2333	6.2	24	2-Furantridecanoic acid, 2,5-dihydro-2-hydroxy-3,4-dimethyl-5-oxo-, methyl ester *	0.22	[84]	
7	29.51	540.3280	C_32_H_46_NO_6_	540.3331	5.1	37.7	Palmerolide A derivative isomer 1 *	1.87	[85,86]	[85,86]
8	29.75	619.2882	C_37_H_39_N_4_O_5_	619.2926	7.1	44	Ethyl pheophorbide A *	5.87	[87]	[46,88]
9	29.93	540.3295	C _32_H _46_NO _6_	540.3331	6.6	15.3	Palmerolide A derivative isomer 2 *	3.93	[85,86]	[85,86]
10	30.22	602.3456	C_32_H_48_N_3_O_8_	602.3487	4.5	37.2	Rhizovarin D *	2.26	[89]	[89]
11	34.95	301.2153	C_20_H_29_O_2_	301.2173	6.6	19.1	Eicosapentanoic acid isomer 1	0.46	[90]	[67,68]
12	35.13	301.2162	C_20_H_29_O _2_	301.2173	3.5	10.5	Eicosapentanoic acid isomer 2	0.47	[90]	[67,68]
13	35.6	227.2013	C_14_H_27_O_2_	227.2017	1.5	3.8	Tetradecanoic acid	1.88	[90]	
14	36.02	253.2166	C_16_H_29_O_2_	253.2173	2.6	33.4	9-Hexadecenoic acid	0.75	[90]	
15	36.22	303.2329	C_20_H_31_O_2_	303.2330	0.1	14.4	Spongian-16-one	5.00	[40]	[40]
16	36.56	279.2323	C_18_H_31_O_2_	279.2330	2.4	9.1	9,12-Octadecadienoic acid	3.84	[90]	[91]
17	36.74	241.2178	C_15_ H_29_O_2_	241.2173	−2.1	4.2	Pentadecanoic acid	5.24	[90]	[47]
18	37.04	283.2637	C_18_H_35_O_2_	283.26.43	2	9.6	Stearic acid	6.66	[90]	[43]
19	37.54	255.2319	C_16_H_31_O_2_	255.2330	2.7	1.5	Hexadecanoic acid isomer 1	0.86	[90]	[46]
20	37.64	331.2640	C _22_H_35_O_2_	331.2643	0.9	11.8	Docosatetraenoic acid isomer 1	3.60	[90]	
21	37.73	331.2646	C_22_H_35_O_2_	331.2643	−1.1	5.1	Docosatetraenoic acid isomer 2	2.76	[90]	
22	37.83	255.2346	C_16_H_31_O_2_	255.2330	−6.6	34	Hexadecanoic acid isomer 2	10.37	[90]	[46]
23	38.16	281.2483	C_18_H_33_O_2_	281.2486	1.2	19.5	9-Octadecenoic acid	2.83	[90]	[46,47]
**Peak**	**RT ^a^**	***m*/*z* experimental**	**Molecular formula (M+H)**	***m*/*z* calculated**	**error (ppm)**	**mSigma**	**Identified compound**	**Area ^b^**	**Identification references**	**Antiproliferative activity**
1	7.21	302.3049	C_18_H_40_NO_2_	302.3054	1.5	3.2	Sphinganine *	1.53	[92]	[73]
2	8.72	482.3604	C_24_H_53_NO_6_P	482.3602	−0.4	31.4	1-O-hexadecyl-sn-glycero-3-phosphocholine (lyso-PAF) *	9.31	[49]	[50,51]

^a^ RT: retention time (minutes). ^b^ Normalized area (%); * Described for the first time in *Phyllidia varicosa* (NA).

**Table 6:** HPLC–ESI–TOF–MS data of the compounds identified in NB extracts in negative and positive ionization mode. BPC is showed in Supplementary Figures 9D and 10D.

**Peak**	**RT ^a^**	***m*/*z* Experimental**	**Molecular Formula (M-H)**	***m*/*z* Calculated**	**Error (ppm)**	**mSigma**	**Identified Compound**	**Area ^b^**	**Identification References**	**Antiproliferative Activity**
1	6.09	218.0820	C_12_H_12_NO_3_	218.0823	1.2	8.5	2,5-Morpholinedione, 3-methyl-6-(phenylmethyl)- *	0.48	[93]	
2	6.81	275.1408	C_15_H_19_N_2_O_3_	275.1401	−2.4	15.8	2,5-Piperazinedione, 3-[(4-hydroxyphenyl)methyl]-6-(2-methylpropyl)- *	0.32	[94]	[95]
3	8.01	255.0894	C_12_H_15_O_6_	255.0874	−7.9	13.3	Phenyl-β-D-galactopyranoside *	0.10	[77]	
4	13.88	454.1905	C_22_H_32_NO_7_S	454.1905	−0.1	23.5	Latrunculol A isomer 1 *	0.55	[96]	[96]
5	14.87	438.1954	C_26_H_24_N_5_O_2_	438.1935	−4.1	18.9	1*H*-Indole, 3-[[3-[5-[4-(4-methyl-1-piperazinyl)phenyl]-2-furanyl]-1,2,4-oxadiazol-5-yl]methyl]- isomer 1	0.58	[97]	
6	15.64	454.1910	C_22_H_32_NO_7_S	454.1905	3.4	37.7	Latrunculol A isomer 2 *	0.15	[96]	[96]
7	15.94	438.1963	C_26_H_24_N_5_O_2_	438.1935	−6.2	37	1*H*-Indole, 3-[[3-[5-[4-(4-methyl-1-piperazinyl)phenyl]-2-furanyl]-1,2,4-oxadiazol-5-yl]methyl]- isomer 2 *	0.24	[97]	
8	17.73	785.3622	C_40_H_49_N_8_O_9_	785.3622	0.8	73.7	Kasumigamide	0.20	[98]	[99]
9	20.15	352.9135	C_9_H_11_Br_2_N_2_O_3_	352.9142	2	359.9	4,5-Dibromo-N-(2,2-dimethoxy-ethyl)-1*H*-pyrrole-2-carboxamide *	8.06	[100]	[100]
10	21.64	601.3731	C_35_H_53_O_8_	601.3746	2.6	12.8	Agosterol E3 *	0.16	[101]	[101]
11	23.8	417.1562	C_22_H_25_O_8_	417.1555	−1.8	2.4	Unknown	0.11		
12	24.45	480.2132	C_23_H_34_N_3_O_6_S	480.2174	8.8	18.1	Carbamic acid, [(1*R*)-1-[[[(acetylamino)methyl]thio]methyl]-2-[[(1*S*)-1-[(acetyloxy)methyl]-2-phenylethyl]amino]-2-oxoethyl]methyl-, 1,1-dimethyl-ethyl ester *	0.37	[102]	
13	26.07	599.2904	C_34_H_39_N_4_O_6_	599.2862	4.9	5.9	Aplysioviolin *	1.01	[102]	[103,104]
14	29.92	540.3353	C_32_H_46_NO_6_	540.3331	−2.1	30.3	Palmerolide A derivative *	0.70	[85,86]	[85,86]
15	34.08	464.3186	C_30_H_42_NO_3_	464.3170	−3.4	27.9	(7Z,10Z,13Z,16Z,19Z)-N-[2-(3,4-Dihydroxyphenyl)ethyl]-7,10,13,16,19-docosapentaenamide *	4.79	[105]	
16	34.95	301.2195	C_20_H_29_O_2_	301.2173	−7.4	10.8	Eicosapentanoic acid	2.59	[90]	[67,68]
17	35.19	277.2195	C_18_H_29_O_2_	277.2173	−8.1	7	Linolenic acid	1.72	[90,106]	[107]
18	35.32	597.4049	C_33_H_57_O_9_	597.4008	−6.9	23.1	Trofoside A	1.89	[108]	[108]
19	35.6	227.2039	C_14_H_27_O_2_	227.2017	−9.8	22.5	Tetradecanoic acid	0.87	[90,106]	
20	35.82	327.2340	C_22_H_31_O_2_	327.2330	−3.1	31.3	Docosahexaenoic acid	0.43	[90,106]	[107]
21	35.85	303.2342	C_20_H_31_O_2_	303.2330	−4.1	24.8	Spongian-16-one isomer 1	0.43	[40]	[40]
22	35.97	253.2189	C_16_H_29_O_2_	253.2173	−6.4	12.2	Palmitoleic acid	1.21	[90,106]	
23	36.22	303.2373	C_20_H_31_O_2_	303.2350	−4.5	19.3	Spongian-16-one isomer 2	9.03	[40]	[40]
24	36.56	279.2353	C_18_H_31_O_2_	279.2330	−8.3	1.1	9, 12-Octadecadienoic acid	7.03	[90]	[91]
25	37.06	283.2663	C_18_H_35_O_2_	283.2643	−7.3	3.7	Stearic acid	2.73	[90]	[43]
26	37.68	331.2670	C_22_H_35_O_2_	331.2643	−8.2	3.6	Docosatetraenoic acid	5.69	[90]	
27	37.94	255.2350	C_16_H_31_O_2_	255.2330	−8.2	16.5	Hexadecanoic acid	5.04	[90]	[46]
28	38.14	281.2508	C_18_H_33_O_2_	281.2486	−8	9.1	9-Octadecenoic acid	4.77	[90]	[46,47]
29	38.91	307.2650	C_20_H_35_O_2_	307.2643	−2.3	6.9	Eicosadienoic acid	0.86	[90,106]	[109]
**Peak**	**RT ^a^**	***m*/*z* experimental**	**Molecular formula (M+H)**	***m*/*z* calculated**	**error (ppm)**	**mSigma**	**Identified compound**	**Area ^b^**	**Identification references**	**Antiproliferative activity**
1	6.89	436.2688	C_24_H_38_NO_6_	436.2694	1.2	4.2	Purpurogemutantin *	3.84	[110]	[110]
2	14.98	535.2694	C_33_H_35_N_4_O_3_	535.2704	1.5	2.4	Pyropheophorbide A *	8.11	[111]	[112]

^a^ RT: retention time (minutes). ^b^ Normalized area (%); * Described for the first time in *Dolabella auricularia* (NB).

## Supplementary Materials

The following are available online at https://www.mdpi.com/2218-273X/9/12/771/s1, Supplementary material and methods, Supplementary Figure S1: dose-response curves showing the effects of 48 h treatment with the CR, PS, NA and NB extracts on the viability of human colon carcinoma cells as assessed using the MTT assay, Supplementary Figure S2: effects of treatment of the CR extract on the proliferation profiles of human colon carcinoma cells, as assessed using the Real Time Cell Analyzer System (RTCA), Supplementary Figure S3: effects of treatment of the PS extract on the proliferation profiles of human colon carcinoma cells, as assessed using RTCA, Supplementary Figure S4: effects of treatment of the NA extract on the proliferation profiles of human colon carcinoma cells, as assessed using RTCA, Supplementary Figure S5: effects of treatment of the NB extract on the proliferation profiles of human colon carcinoma cells as assessed using RTCA, Supplementary Figure S6: effects of marine extracts on the formation of colonies by HGUE-C-1, HT-29 and SW-480 cells, Supplementary Figure S7: marine extracts alter the mitochondrial membrane polarization (MMP) in colon cancer cells, Supplementary Figure S8: marine extracts cause mitochondrial membrane depolarization (MMD) in colon cancer cells, Supplementary Figure S9: base peak chromatograms (BPCs) of the four extracts in negative ionization mode, Supplementary Figure S10: base peak chromatogram (BPCs) of the four extracts in positive ionization mode, Supplementary Table S1: yield rate (%) with solid of complete marine extracts, Supplementary Table S2: IC_50_ values from MTT assay of complete extracts cytotoxic effect in 3 different colon cancer cell lines.
